# Understanding Sorption of Aqueous Electrolytes in Porous Carbon by NMR Spectroscopy

**DOI:** 10.1021/jacs.3c14807

**Published:** 2024-04-01

**Authors:** Dongxun Lyu, Katharina Märker, Yuning Zhou, Evan Wenbo Zhao, Anna B. Gunnarsdóttir, Samuel P. Niblett, Alexander C. Forse, Clare P. Grey

**Affiliations:** Yusuf Hamied Department of Chemistry, University of Cambridge, Cambridge CB2 1EW, United Kingdom

## Abstract

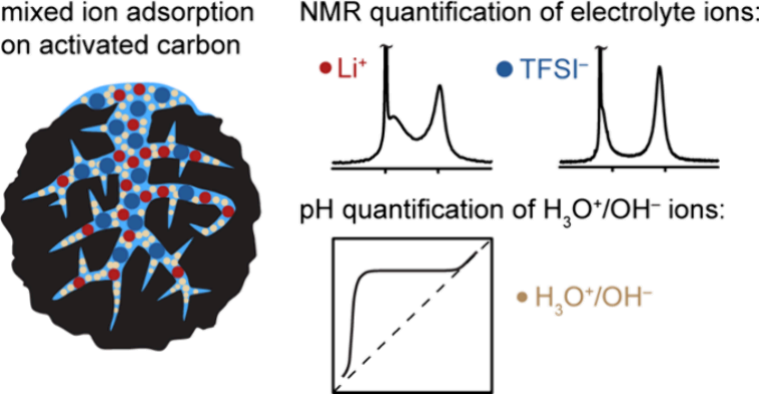

Ion adsorption at solid–water interfaces is crucial for many electrochemical processes involving aqueous electrolytes including energy storage, electrochemical separations, and electrocatalysis. However, the impact of the hydronium (H_3_O^+^) and hydroxide (OH^–^) ions on the ion adsorption and surface charge distributions remains poorly understood. Many fundamental studies of supercapacitors focus on non-aqueous electrolytes to avoid addressing the role of functional groups and electrolyte pH in altering ion uptake. Achieving microscopic level characterization of interfacial mixed ion adsorption is particularly challenging due to the complex ion dynamics, disordered structures, and hierarchical porosity of the carbon electrodes. This work addresses these challenges starting with pH measurements to quantify the adsorbed H_3_O^+^ concentrations, which reveal the basic nature of the activated carbon YP-50F commonly used in supercapacitors. Solid-state NMR spectroscopy is used to study the uptake of lithium bis(trifluoromethanesulfonyl)-imide (LiTFSI) aqueous electrolyte in the YP-50F carbon across the full pH range. The NMR data analysis highlights the importance of including the fast ion-exchange processes for accurate quantification of the adsorbed ions. Under acidic conditions, more TFSI^–^ ions are adsorbed in the carbon pores than Li^+^ ions, with charge compensation also occurring via H_3_O^+^ adsorption. Under neutral and basic conditions, when the carbon’s surface charge is close to zero, the Li^+^ and TFSI^–^ ions exhibit similar but lower affinities toward the carbon pores. Our experimental approach and evidence of H_3_O^+^ uptake in pores provide a methodology to relate the local structure to the function and performance in a wide range of materials for energy applications and beyond.

## Introduction

The formation and modulation of the electric double layer on a surface or in a pore represent key mechanisms that underpin the operation of a surprisingly large range of modern technologies and processes, including supercapacitors, water desalination, electrocatalysis, and electrochemical CO_2_ capture.^1−4^ Of the materials and interfaces that enable these technologies, the porous carbon electrode–water interface is particularly important, since porous carbon electrodes provide large (internal) surface areas for energy storage, ion separation, and electrocatalysis. Aqueous electrolytes are ideal for large-scale electrochemical systems, enabling easier device construction while also being environmentally benign. However, the role of the water at these electrochemical interfaces, especially at different pH levels, has been underexplored, in part due to the difficulties in characterizing and theoretically modeling water-based electrolyte/electrode interactions; furthermore, the role of oxygen-containing functional groups in modifying these interactions is rarely considered.

A range of studies have highlighted the importance of water and hydronium species in electrochemical systems. A recent study of the electrode material birnessite in aqueous electrolytes demonstrated the importance of interlayer water molecules in mediating the interaction between electrolyte ions and the birnessite host, which ultimately controls the capacitance.^5,6^ Luo et al. reported an imbalance of anions and cations in nanoconfined aqueous sodium salt electrolytes as observed by nuclear magnetic resonance (NMR) spectroscopy, which was ascribed to the less negative hydration enthalpy of anions over Na^+^ cations, known as the specific ion effect.^7^ Meanwhile, recent electrochemical quartz crystal microbalance (EQCM) studies of a carbide-derived carbon-based supercapacitor with an EMI^+^-HSO_4_^–^ (1-ethyl-3-methylimidazolium bisulfate) electrolyte (pH = 0.8) illustrated that the main charge carrier responsible for charge compensation at the negative potentials was in fact H^+^, rather than the bulkier EMI^+^ ions, based on the negligible measured mass changes observed for the negative electrode.^8^ These studies highlight the role of the water molecules in aqueous electrolytes, especially in the ionized form as hydronium (H_3_O^+^) and hydroxide (OH^–^) ions. In principle, the H_3_O^+^ and OH^–^ ions can compete with electrolyte salt ions to carry charges across an electrochemical device and serve as charge-compensating species at the interfaces. Electric double layer theory predicts that the electronic charge of a solid electrode is balanced by a redistribution of counterions in aqueous electrolytes; however, it is unclear whether and how H_3_O^+^ and OH^–^ ions contribute to the formation and stabilization of the interfacial electric double layer. To understand this process, the composition of the charge-compensating species and how they evolve under a range of pH conditions inside carbon nanopores should be determined.

It is extremely challenging to experimentally obtain a molecular-level picture of ion distributions at the electrode–electrolyte interfaces, which is in part due to the difficulty of tracking individual types of charged species as they undergo fast ion dynamics in aqueous electrolytes. Studies of the interfacial ion adsorption in carbons are further complicated by their hierarchical and disordered structures and the presence of nontrivial concentrations of oxygen-containing functional groups, the latter also potentially contributing to charge storage mechanisms.^9^ Given that the ions that are adsorbed into the carbon pores are the ones responsible for charge and, thus, energy storage,^10,11^ quantification of in-pore ion adsorption is crucial for understanding the electrode–electrolyte interfaces. To this end, NMR spectroscopy has been widely used to study the adsorption of molecules and ions in porous carbons since it is well established that resonances corresponding to species adsorbed inside carbon pores show a shift to lower frequencies relative to the frequency of the bulk species.^12−18^ Particularly relevant to this study are the recent NMR studies of aqueous electrolytes by Cervini et al.^18^ who studied polyether–ether–ketone (PEEK)-derived activated carbons (PDCs), along with the earlier study of Luo et al. also on PDCs.^7^

Here, we study the uptake of an aqueous lithium bis(trifluoromethanesulfonyl)imide (LiTFSI) electrolyte in the commercial activated carbon “YP-50F” with NMR spectroscopy. pH measurements are first performed to quantify H_3_O^+^ ion adsorption, the results showing that the porous carbon studied here has a very basic point of zero charge (PZC), indicating strong basicity on the carbon surface. By using a combination of X-ray photoelectron spectroscopy (XPS) and acid–base titration, we then identify and quantify the functional groups in the carbons that likely contribute to this PZC. The NMR experiments reveal rapid exchange between ions in the bulk electrolyte and in carbon pores, these fast exchange processes having a substantial effect on the apparent concentration of adsorbed Li^+^ and TFSI^–^ ions as quantified by NMR. To address this, we establish a quantification method that involves deconvolution of the spectra based on a two-site exchange model. Equipped with this NMR method, we show that in acidic electrolytes, there is an apparent imbalance between the adsorption of TFSI^–^ ions and Li^+^ ions when H_3_O^+^ adsorption is neglected. By combining pH measurements (H_3_O^+^ adsorption) with NMR spectroscopy (TFSI^–^ and Li^+^ adsorption) and analysis of surface functional groups, we obtain a more quantitative picture of the ion adsorption and show that the significant uptake of H_3_O^+^ in these carbons with acidic electrolytes must be accounted for to understand sorption in these materials. Our experiments indicate that the carbon pores become more ionophilic as the electrolyte becomes more acidic, which has important implications for the charge storage mechanisms of aqueous electrochemical systems; indeed, significant increases in the capacitance of YP-50F carbon are observed experimentally here when using acidic electrolytes.

## Experimental Section

### Activated Carbon

YP-50F (Kuraray Chemical, Japan) activated carbon was thoroughly rinsed with deionized water (Millipore) to remove any residual KOH salt left from the activation process until the pH of the water used in the washing became neutral. The washed carbon was dried in vacuo at 100 °C for at least 48 h before use.

### YP-50F Carbon Film

For ease of handling, a free-standing YP-50F carbon film was prepared for the XPS measurements and electrochemical characterization by mixing YP-50F carbon powder (95 wt %) with polytetrafluoroethylene (PTFE) binder (5 wt %) (Sigma-Aldrich, 60 wt % dispersion in water) in ethanol. The resulting film was rolled to give a carbon film of approximately 0.25 mm in thickness.

### Pore Size Distribution

The carbon pore size distribution was obtained by analyzing N_2_ gas sorption isotherms recorded on dried YP-50F activated carbon powder at 77 K using an Autosorb iQ from Quantachrome Instruments. Prior to analysis, in situ degassing (80 °C, 24 h) was performed on a Micromeritics VacPrep. The Brunauer–Emmett–Teller (BET) surface area was calculated from the isotherm using the BET equation and Rouquerol’s consistency criteria implemented in BET surface identification (BETSI).^19^ The micropore volume (*V*_micro_) and total pore volume (*V*_total_) were calculated at a relative pressure (*P*/*P*_0_) ranging from 0.1 to 0.99. For YP-50F, a type I N_2_ isotherm was observed, with high gas uptake below 0.1 *P*/*P*_0_ indicating extensive microporosity.

### NMR Samples

A 1 M LiTFSI aqueous electrolyte was prepared by dissolving lithium bis(trifluoromethanesulfonyl)imide (LiTFSI) salt (Sigma-Aldrich, >99.95% purity) in deionized water (Millipore). For YP-50F partially filled with an electrolyte, 10 μL of 1 M LiTFSI aqueous electrolyte was pipetted into a 4 mm NMR rotor containing 20 mg of dried YP-50F carbon powder to generate a 0.5:1 volume of electrolyte/weight of carbon (v/w) ratio. The rotor was then tightly sealed and left for 24 h for the adsorption to reach equilibrium. For YP-50F saturated in the electrolyte, 30 μL of 1 M LiTFSI aqueous electrolyte was injected into 10 mg of dried YP-50F carbon powder to produce 3:1 v/w.

For the H_3_O^+^ adsorption studies, five acidic LiTFSI mixtures were prepared by mixing 1 M HTFSI acid with a 1 M LiTFSI electrolyte in volumetric ratios of 50:50, 40:60, 30:70, 20:80, and 10:90, giving LiTFSI electrolytes pH of 0.51, 0.54, 0.59, 0.74, and 1.05, respectively. Three basic LiTFSI mixtures were prepared by mixing 1 M LiOH base with a 1 M LiTFSI electrolyte in volumetric ratios of 50:50, 30:70, and 10:90, giving LiTFSI electrolytes pH of 11.48, 11.46 and 11.40, respectively. The reported pH of the LiTFSI electrolyte mixtures was determined using a pH meter (905 Titrando, Metrohm) while purging the mixtures under Ar. For each individual LiTFSI electrolyte, 30 μL of LiTFSI electrolyte mixture was pipetted into 10 mg of dried YP-50F carbon powder in a 4 mm rotor with a 3:1 v/w ratio. As before, the rotor was then tightly sealed and left for 24 h to reach equilibrium.

To study the effect of concentration, 0.50, 0.75, and 1 M LiTFSI aqueous electrolytes were prepared. Each electrolyte (30 μL) was injected into 10 mg of dried YP-50F carbon powder at a ratio of 3:1 v/w, resulting in three carbon samples with different LiTFSI salt concentrations.

### NMR Experiments

Solid-state NMR experiments were performed at three magnetic field strengths: a Bruker Avance II spectrometer operating at a magnetic field strength of 4.7 T (200 MHz ^1^H Larmor frequency) using a Bruker 4 mm HX double-resonance probe, a Bruker Avance spectrometer at 7.1 T (300 MHz ^1^H Larmor frequency) using a Bruker 2.5 mm HX double-resonance probe, and a Bruker Avance III spectrometer at 16.5 T (700 MHz ^1^H Larmor frequency) using a Bruker 4 mm HXY triple-resonance probe. All spectra were acquired using a simple one pulse-acquire sequence at a MAS frequency of 5 kHz, and radiofrequency (rf) pulses were applied at ∼120 kHz rf field strength for ^7^Li, ∼96 kHz for ^19^F, and ∼100 kHz for ^1^H. One hundred and twenty-eight transients were accumulated for each ^7^Li spectrum, 64 transients for each ^19^F spectrum, and 32 transients for each ^1^H spectrum. Recycle delays of 60, 30, and 15 s were used in the ^7^Li, ^19^F, and ^1^H experiments for quantitative analysis, respectively. ^7^Li chemical shifts were referenced externally using lithium carbonate (Li_2_CO_3_) at 0 ppm, and ^1^H chemical shifts were referenced relative to adamantane (C_10_H_16_) at 1.9 ppm. ^19^F chemical shifts were referenced relative to 1 M LiTFSI at 295 K at −78.6 ppm since the ^19^F chemical shift is temperature-dependent. Deconvolution of the ^7^Li, ^19^F, and ^1^H spectra was carried out using Dmfit software, and an example of the deconvolution is given in the Supporting Information. For variable-temperature NMR measurements, the temperature was previously calibrated by using the temperature-dependent chemical shift of ^79^Br in potassium bromide (KBr) and the frictional heating effect at 5 kHz MAS is less than 2 °C.^12^

### Two-Site Exchange NMR Model

The chemical exchange of the ^7^Li and ^19^F NMR spectra was simulated based on a two-site exchange model and using an expression derived by Norris.^20^ Details of the simulation can be found in the Supporting Information.

### XPS

The XPS measurement was performed on a YP-50F carbon film with a monochromatic Al Kα X-ray source (*h*ν = 1486.6 eV) using a SPECS PHOIBOS 150 electron energy analyzer with a total energy of 500 meV.

### Boehm Titration

Acid–base Boehm titrations^21^ were performed to quantify the acidic and basic functional groups on the surface of YP-50F carbon by using the following procedure: 50 mL of either 0.05 M NaOH, Na_2_CO_3_, or NaHCO_3_ was added to 1.5 g of YP-50F carbon powder to quantify the acidic functional groups; 50 mL of 0.05 M HCl electrolyte was added to 1.5 g of YP-50F carbon powder to quantify the basic functional groups. Each suspension was sealed and stirred with a magnetic stirrer at 500 rpm for 24 h. The mixture was filtered to remove the YP-50F carbon, and a 10 mL aliquot was taken out from each aqueous electrolyte. The basic aliquots (NaOH, Na_2_CO_3_, and NaHCO_3_) were acidified by adding 30 mL of 0.05 M HCl to ensure complete neutralization of the base. The acidified electrolytes were then back-titrated with 0.05 M NaOH electrolyte under an Ar atmosphere using a 905 Titrando (Metrohm) autotitrator. The aliquot taken from the acidic (HCl) sample was titrated directly with 0.05 M NaOH electrolyte. The amount of the acidic and basic functional groups was then calculated based on the assumptions that NaHCO_3_ can only neutralize the carboxylic functional groups, Na_2_CO_3_ neutralizes carboxylic and lactonic functional groups, NaOH neutralizes carboxylic, lactone, and phenol functional groups, and HCl neutralizes all the basic functional groups. The amount of acidic groups on the carbons was determined by
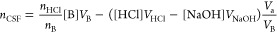
1where [B] and *V*_B_ are the concentration (in M) and volume (in L) of the base mixed with the carbon, respectively, *n*_CSF_ denotes the moles of carbon surface functionalities that reacted with the base during mixing, *V*_a_ (L) is the volume of aliquot taken from *V*_B_ (L), and [HCl] and *V*_HCl_ are the concentration (M) and volume of the HCl acid (L) used to acidify the aliquots, respectively.

### H_3_O^+^ Adsorption Experiments

**Figure 1:**
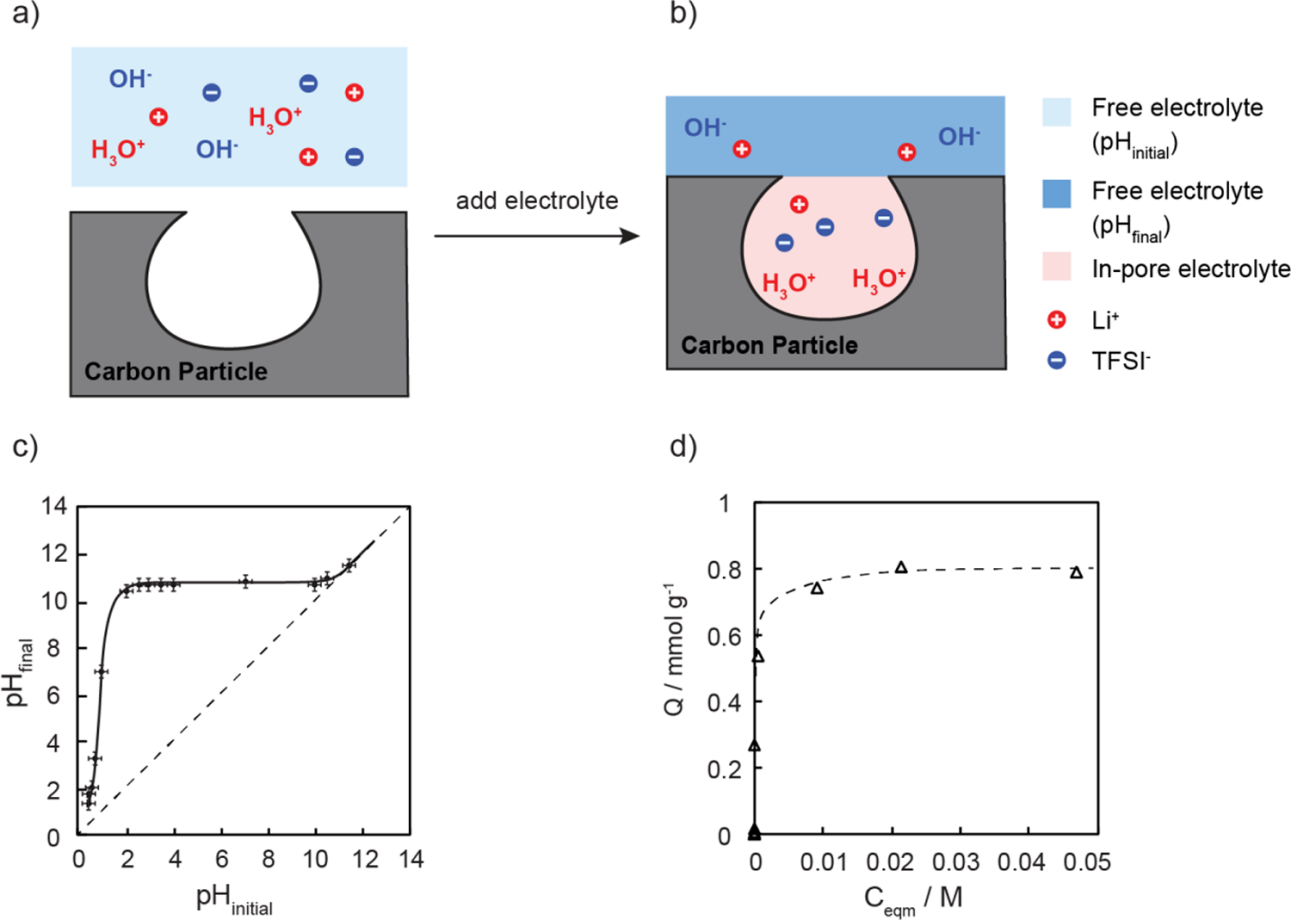
(a) Illustration of the ion adsorption process involving the aqueous LiTFSI electrolyte before (a) and after (b) it adsorbed into the carbon pores. The pH of the electrolyte before immersing with the carbon is labeled “pH_initial_”; after pore filling, the pH of the remaining electrolyte outside of carbon particles is labeled “pH_final_”. (c) Plot of pH_initial_ vs pH_final_ of YP-50F carbon soaked with a 1 M LiTFSI aqueous electrolyte over the full pH range. The solid line is a guide to the eye. The diagonal dashed line represents the pH if no H_3_O^+^or OH^–^ adsorption occurs, i.e., pH_initial_ = pH_final_. (d) H_3_O^+^ adsorption data plotted as *Q* (H_3_O^+^ adsorption capacity in mmol g^–1^) vs *C*_eqm_ (concentration of H_3_O^+^ left in the bulk electrolyte at equilibrium in mol L^–1^); the data was fitted to an H-type adsorption model (shown as the dashed line) to extract the maximum adsorption capacity.

The H_3_O^+^ adsorption experiments shown in Figure 1c,d were carried out by adding 1 g of the washed YP-50F activated carbon into 3 mL of a series of 1 M LiTFSI aqueous stock electrolytes each prepared at a different pH value ranging from 0.5 to 12. The pH value of each stock electrolyte was adjusted by adding either degassed 1 M LiOH electrolyte or degassed 1 M HTFSI electrolyte as appropriate. The resulting carbon suspensions were stirred at 500 rpm at a constant temperature (22 °C) for 48 h to reach equilibrium and then centrifuged at 3000 rpm.

The equilibrium pH of the 1 M LiTFSI electrolyte after adding in carbon was measured using a pH meter (905 Titrando, Metrohm) under N_2_ at 293 K. The equilibrium H_3_O^+^ uptake *Q* (mmol g^–1^) was calculated from the equation:
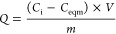
2where *V* is the electrolyte volume (in L), *C*_i_ is the initial H_3_O^+^ concentration (mmol L^–1^), *C*_eqm_ is the equilibrium H_3_O^+^ concentration (mmol L^–1^), and *m* is the mass of the dried YP-50F activated carbon (g).

### Electrochemical Characterization

The two-electrode electrochemical characterization of YP-50F carbon was performed using 2032 coin cells (Cambridge Energy Solution) with the LiTFSI electrolyte under acidic, neutral, and basic conditions. The acidic electrolyte was composed of a 50:50 mixture of 1 M HTFSI and 1 M LiTFSI electrolytes, resulting in a pH of 0.5, the neutral electrolyte was composed of 1 M LiTFSI electrolyte, with a pH of 7.03, and the basic electrolyte comprised a 50:50 mixture of 1 M LiOH and 1 M LiTFSI electrolytes, giving a pH of 12. The coin cells consisted of two 14 mm YP-50F carbon film electrodes (fabricated as previously described), a 16 mm glass fiber separator (Whatman, GF/A), and 80 μL of 1 M LiTFSI aqueous electrolytes.

Cyclic voltammetry measurements were performed between 0 and 1 V at a scan rate of 0.5 mV/s. Capacitances were measured using galvanostatic charge–discharge experiments at a mass-normalized constant current of 0.2 mA/g. The gradient of the discharge slope (, V s^–1^) was extracted to calculate the capacitance using eq 3:
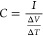
3where *I* is the normalized constant charging/discharging current (A/g).

### Density Functional Theory Calculations

All geometry optimizations and NMR calculations were performed using Gaussian 03 software.^22^ Geometries were optimized using the B3LYP exchange correlation functional with the 6-31G(d) basis set.^23^ Previous work showed that with different basis sets, the variation in magnetic field calculated near aromatic carbon rings is minimal.^24^

## Results

### Quantifying H_3_O^+^ Adsorption in Activated YP-50F via a pH Study

Since H_3_O^+^, OH^–^, and H_2_O cannot be differentiated readily by using ^1^H NMR spectroscopy due to their fast exchange with each other, the H_3_O^+^ uptake in YP-50F was first examined and quantified via the pH changes of the LiTFSI electrolyte before (pH_initial_) and after adding in YP-50F carbon (pH_final_), as schematically illustrated in Figure 1a,b. A series of LiTFSI–HTFSI/LiOH electrolyte mixtures were first prepared over a wide range of pH such that the H_3_O^+^ concentration was varied without introducing any new anion or cation to the aqueous LiTFSI electrolyte. The concentration of adsorbed H_3_O^+^ was then quantified via the pH change observed on addition of a known amount of the YP-50F carbon (1 g) to 3 mL of an electrolyte mixture at pH_initial_ (Figure 1c). For example, an initial pH (pH_initial_) of 1.05 was measured for the 1 M LiTFSI electrolyte mixed with 10% HTFSI acid (v/v). After adding the YP-50F carbon, the pH of the liquid suspension increased significantly to 6.89 (pH_final_), indicating that the carbon is basic and that it adsorbs H_3_O^+^ ions. A point of zero charge (PZC), defined as the pH value of the electrolyte when the net charge on the surface is zero under given conditions of temperature and pressure,^25^ of 10.6 ± 0.2 was determined from the intercept between the pH sorption plateau and the diagonal line of pH_initial_ = pH_final_. The pH changes were then converted into H_3_O^+^ uptake by accounting for the volume of the added electrolyte, the pH sorption data, and the relationship between the amount of H_3_O^+^ adsorbed in the carbon (*Q*) and the amount of H_3_O^+^ remaining in the liquid phase (*C*_eqm_), as plotted in Figure 1d. The maximum H_3_O^+^ adsorption capacity of YP-50F was determined from the plateau to be 0.8 mmol g^–1^ for the 1 M LiTFSI/HTFSI electrolyte.

### Study of Surface Functional Groups on and in YP-50F Carbons

To explore the origin of the observed H_3_O^+^ uptake, the nature of the surface functional groups on YP-50F was examined by both Boehm acid–base titration and XPS measurements.^26,27^ A Boehm acid–base titration enables determination of both internal and external oxygen-containing functionalities (Table 1): the weakest base NaHCO_3_ neutralizes the most acidic species, namely, the carboxylic groups, yielding 0.023 ± 0.002 mmol g^–1^ carboxylic groups, the second weakest base Na_2_CO_3_ neutralizes weaker acids such as lactones in addition to carboxylic groups,^28^ and a lactone concentration of 0.015 ± 0.005 mmol g^–1^ is determined. The strongest base NaOH allows quantification of all the acidic groups including the weakly acidic phenols, yielding a phenol (Ph–OH) concentration of 0.205 ± 0.005 mmol g^–1^, and a total concentration of acidic functional groups of 0.243 ± 0.015 mmol g^–1^. The basic functional groups such as chromenes and pyrones (species generally thought to be present in activated carbons^29^) were quantified by HCl titration (using a pH 1, 0.05 M HCl solution), yielding 1.20 ± 0.01 mmol g^–1^, which is comparable to the 1.12 mmol g^–1^ reported in the literature for YP-50F carbon.^30,31^

**Table 1:** Summary of the Different Oxygen-Containing Functional Groups in YP-50F Carbon As Determined by XPS, Boehm Titration, and pH Measurements

		**acidic functional group** (mmol g^–1^ or atomic %)			**basic functional group** (mmol g^–1^ or atomic %)	
	**oxygen content =** (atomic %)	carboxylic	lactone	phenol	chromene, pyrone	**total H_3_O^+^uptake** (in 1 M LiTFSI, mmol g^–1^)
Boehm titration (current study)	not applicable, N/A	0.023 ± 0.005	0.015 ± 0.005	0.205 ± 0.005	1.20 ± 0.005	(N/A)
Boehm titration (literature)^30^	N/A	0.002	0.028	0.232	1.132	N/A
XPS (current study)	5.2%	1.8%		1.4%	2.0%	N/A
XPS (literature)^32^	5.3%	5.3%				N/A
pH study (current study)	N/A	N/A				0.8

Of particular relevance to this study, the concentration of basic groups measured via Boehm titration using 0.05 M HCl (1.2 mmol g^–1^) is higher than the H_3_O^+^ uptake of 0.8 mmol g^–1^ determined by the H_3_O^+^ adsorption measurements described above (specifically performed at pH 0.5 using 3 mL of a 50:50 acidic mixture of 1 M LiTFSI and 1 M HTFSI and 1 g of carbon). However, the concentration of H_3_O^+^ in 3 mL of this 50:50 mixture is not sufficient to titrate all the basic groups in the carbon powder, in part accounting for the lower concentration of H_3_O^+^ uptake. This motivated a more detailed study of both the effect of salt concentration and the nature of the ions on the PZC and ion uptake. Further titration measurements were performed (see Supporting Information Section 1), and a higher PZC of 11.0 was measured when performing the PZC measurement with a 0.01 M series of LiTFSI/HTFSI/LiOH electrolytes (Figure S1), i.e., a higher concentration of basic sites appears to be present at low salt concentrations. As discussed further below, this suggests that as the salt concentration increases, the Li^+^ cations will increasingly start to compete with the H_3_O^+^ to charge-compensate the negatively charged functional groups, resulting in a lower measured uptake of H_3_O^+^. Furthermore, a decrease in PZC is seen when using nitrate solutions, highlighting the role that anion uptake and p*K*_a_ of the acidic groups play in controlling sorption processes.

The carbon surface functionality was further characterized by XPS (Table 1 and Figure S2), determining the elemental composition on the outermost surface layers of the carbon particles (i.e., up to 5 nm depth). Analysis of the C 1s region indicates that there are five types of carbon functionalities present in the YP-50F carbon film, namely, the hydrocarbons (C–C/C–H groups at 284.9 eV), phenols or ethers (C–OH/C–O–C at 285.9 eV), carbonyls (C=O at 287.3 eV), carboxylic acids or esters (O–C=O at 289.5 eV), and PTFE binder (C–F at 291.2 eV).^26,33,34^ The O 1s XPS spectrum is consistent with the analysis of the C 1s XPS spectrum, with three peaks being observed that are assigned to carbonyl groups (O=C at 531.5 eV), phenols or esters (C–OH/C–O–C at 532.5 eV), and carboxylic groups (O=C–OH at 533.5 eV).^26,33,34^ A percentage of oxygen-containing groups on the carbon surfaces of 5.2% (by mole) is obtained by determining their contribution to the total area under the C 1s curve after subtracting the binder contribution (C–F peak) (Table 1). This value agrees well with the oxygen content of 4.4% for YP-50F in the literature, i.e., a C:O ratio of 1:18.^26^ Furthermore, the analysis of the relative intensity of the different peaks in the XPS data suggests that the outermost surface of YP-50F consists of 3.2% acidic groups and 2.0% basic groups and, thus, the external surface is slightly acidic. By contrast, the Boehm titration measurements support the idea that bulk YP-50F has more accessible basic groups than acidic groups on its external and internal surfaces, which is consistent with the PZC obtained for YP-50F of 10.6 ± 0.2 and the measured total H_3_O^+^ uptake of 0.8 mmol g^–1^ when 1 g of carbon is added to 3 mL of an electrolyte.

### NMR Study of Adsorption of an Aqueous 1 M LiTFSI Electrolyte in YP-50F

**Figure 2:**
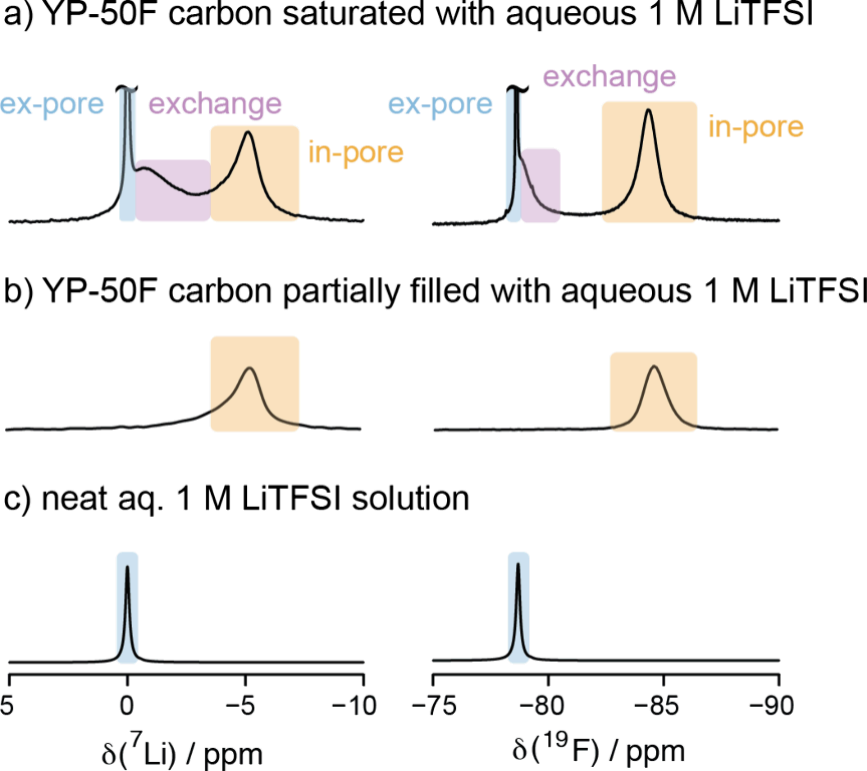
^7^Li and ^19^F NMR MAS spectra (7.1 T, MAS = 5 kHz) of (a) YP-50F carbon saturated with a 1 M LiTFSI aqueous electrolyte at a ratio of 1:3 w/v; (b) YP-50F carbon partially filled with a 1 M LiTFSI aqueous electrolyte at a ratio of 1:0.5 w/v. (c) ^7^Li and ^19^F NMR static spectra of a neat 1 M LiTFSI aqueous electrolyte with no added YP-50F carbon. The free or “ex-pore” ions (“ex-pore”, blue), ions in the micropores (“in-pore”, yellow), and ions exchanging between the in-pore and ex-pore electrolytes (“exchange”, lilac) are highlighted.

To quantify the salt uptake in nanoporous carbons, the ^19^F and ^7^Li NMR spectra were acquired for YP-50F carbon powder soaked with different amounts of aqueous 1 M LiTFSI electrolyte and three resonances are observed (Figure 2a). In an attempt to assign the NMR spectroscopic features, these spectra are compared with the spectra of YP-50F carbon partially filled with the LiTFSI electrolyte (Figure 2b) and the neat LiTFSI aqueous electrolyte (Figure 2c). The sharp ^7^Li (at 0.4 ppm) and ^19^F (at −78.6 ppm) signals only appear in the neat LiTFSI aqueous electrolyte and in the fully filled carbon slurry, and thus, these signals can be assigned with confidence to the ions occupying large reservoirs of electrolyte between primary carbon particles (referred to as “ex-pore” ions highlighted in blue) diffusing freely, as in the bulk electrolyte. Slightly broader ^7^Li and ^19^F NMR resonances were detected at respective chemical shifts of −4.9 and −84.2 ppm for both the partially filled and saturated carbons, and these resonances are assigned to ions in the nanometer-sized pores of the carbon (“in-pore” ions, highlighted in yellow). The separation between the in-pore and ex-pore resonance, Δδ, has values of −5.6 ppm for ^19^F NMR and −5.3 ppm for ^7^Li NMR, which are similar to the reported values of −6.3 and −6.5 ppm for TFSI anions in EMI TFSI ionic liquid and 1.5 M LiTFSI acetonitrile electrolytes adsorbed in YP-50F carbon, respectively.^12,35^ These shifts are due to the local magnetic field arising from delocalized π-electrons in the predominantly sp^2^-bonded carbon surfaces, also known as the ring current effect.^10,36^ The similar in-pore chemical shifts observed in both ^7^Li and ^19^F NMR spectra are consistent with the idea that the ring current shift is nucleus-independent to the first approximation; the small differences may reflect the differences in the average ion–carbon distances but may also be due to dynamics (see later).^35,36^ The slightly smaller Δδ values for the aqueous vs non-aqueous electrolytes are tentatively attributed to the differences in the hydration shells of these ions, along, potentially, with interactions with functional groups, as discussed below. Finally, the resonances located in between the “in-“ and “ex-“ pore peaks at −1 and −79 ppm in ^7^Li and ^19^F NMR spectra, respectively, are assigned to the ions exchanging rapidly between the bulk and in-pore environments. A similar assignment of the “exchange” peak in the ^1^H NMR spectra of water molecules in pure water adsorbed in a PEEK-derived activated carbon was recently reported by Cervini et al.^18^ The assignments are further supported by two-dimensional exchange (EXSY) spectra, where cross peaks are observed between ex-pore and exchange peaks and between exchange and in-pore peaks (see Supporting Information Section 6).

### Variable Temperature NMR Spectroscopy as a Probe of In- and Ex-Pore Exchange

**Figure 3:**
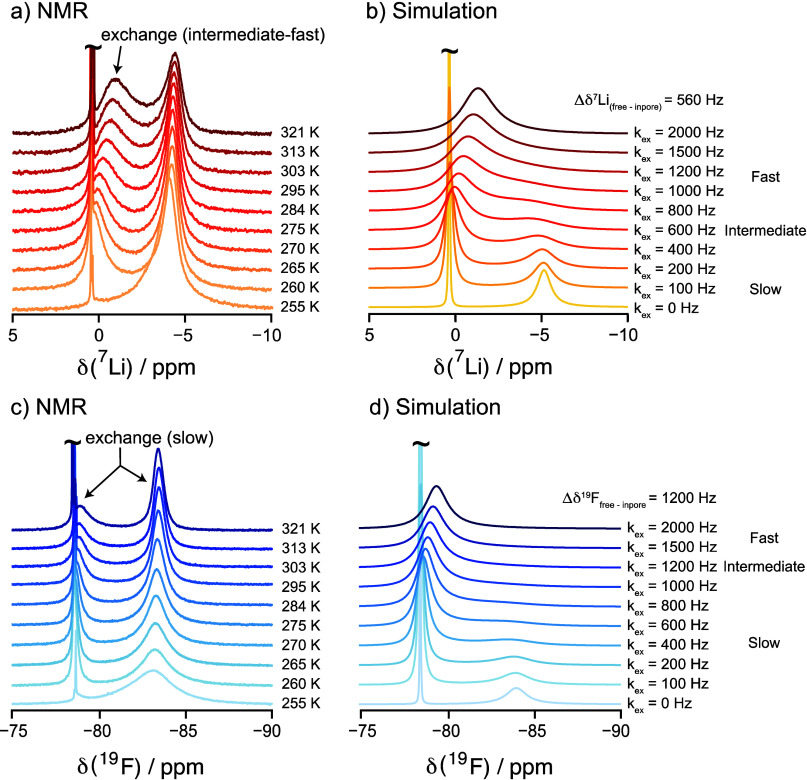
Variable temperature experiments of YP-50F carbon saturated with a 1 M LiTFSI aqueous electrolyte. (a) ^7^Li and (c) ^19^F MAS NMR spectra (7.1 T, MAS = 5 kHz) were recorded from 321 to 255 K. All of the ex-pore electrolyte peaks in the experimental spectra are truncated to show the in-pore region more clearly. Simulated spectra (^7^Li, b, and ^19^F, d) performed using a simple two-site exchange model to represent the exchange between ex-pore and in-pore ions. A single correlation time (and, thus, exchange frequency) was assumed. Simulations using frequencies between 0 and 2000 Hz and ratios for the ex-pore to in-pore populations of 6:1 and 1.5:1 for the ^19^F and ^7^Li spectra, respectively, are shown.

To explore the origin of the exchange resonances observed in Figure 2, ^7^Li and ^19^F NMR spectra of the YP-50F carbon saturated with a 1 M LiTFSI aqueous electrolyte were recorded over a range of temperatures (Figure 3). For the ^7^Li NMR spectra in Figure 3a, on cooling the sample from 321 to 260 K, the in-pore signal increases in intensity and the “exchange” resonance displays a gradual decrease in linewidth and intensity while also shifting toward the ex-pore electrolyte resonance. At 255 K, the “exchange” peak has disappeared completely, with no evidence for exchange, likely due to a combination of the electrolyte freezing and a more sluggish exchange. The disappearance of the exchange peak at 255 K is accompanied by an increase in intensity of the in-pore resonance. Despite the change in in-pore signal intensity with temperature, the in-pore Li^+^ ion population remains essentially constant with temperature when taking into account the increase in in-pore ion contribution from the exchange peak (Figure S4). These changes of line shape and signal intensities with temperature are fully reversible (Figure S5). This spectral behavior (at least above the electrolyte freezing point) is consistent with the well-established chemical exchange phenomenon in NMR spectroscopy, confirming that this middle peak arises from electrolyte ions undergoing chemical exchange. For the ^19^F NMR spectra in Figure 3c, on cooling the sample from 321 to 260 K, the in-pore signal decreases in intensity but broadens in linewidth, and the “exchange” resonance gets narrower at lower temperatures until it disappears completely at 255 K. The chemical shift of the ^19^F “exchange” resonance barely changes across the temperature range, unlike the shift seen for this resonance in the ^7^Li spectra. Note that the relatively sharp peak of the TSFI^–^ resonance, even at 260 K, indicates that these ions are mobile inside the carbon pores, well below the freezing point of water. Furthermore, the concentration of ions in the pores has increased as the water outside the pores freezes.

To understand the evolution of the “exchange” resonance across the temperature range, the ^7^Li and ^19^F spectra of the ions undergoing exchange processes were simulated. While being overly simple, we assumed a two-site exchange model with the ions moving (hopping) in and out of the pores with a range of exchange rates (*k*_ex_) from 0 to 2000 Hz for ^7^Li and ^19^F (Figure 3b,d). We have not included the subset of ions that are not in rapid exchange, i.e. (a) in the bulk electrolyte, but too far from the subset of ex-pore ions that are in fast exchange with the in-pore ions, and (b) in the pores but too distant from the surfaces of the carbon so that they cannot undergo rapid exchange with the ex-pore ions.

Despite its simplicity, the exchange model qualitatively reproduces the exchange peak of ^7^Li spectra across the whole temperature range (Figure 3a,b), the simulations demonstrating that the exchange between ex-pore and in-pore Li^+^ ions is in the “intermediate-to-fast” exchange regime at room temperature. The simulations reproduce the notable shift of the exchange peak that is observed as a function of temperature, in addition to the motional narrowing of the exchange peak seen above 303 K, both observations being consistent with the coalescence of the peaks from a subset of in- and ex-pore Li^+^ ions. It is, however, important to note that while the experimental ^7^Li spectra contain an exchange peak consistent with the fast-intermediate exchange, this resonance does not represent all the in-pore Li^+^ ions in the sample (note assumption made above), since a clear in-pore peak (in the slow exchange regime) is also observed even at the highest temperature studied. The spectra reflect a distribution of exchange rates between Li^+^ ions in different chemical environments within the pores of the sample. The carbon powder has a distribution of particle sizes (ranging from 2 to 10 μm) and pore sizes (85% of pores are below 2 nm, see Figure S3), and there are multiple pathways and distances that the ions must travel to move in and out of the carbon pore structures. Thus, there will not be a single discrete exchange rate, and a distribution of exchange rates within a certain range would be a better description of the ion dynamics in these systems. Consistent with this, the ^7^Li EXSY experiments (Figure S7), performed with a 1 ms mixing time, show that on this time frame, the in-pore and exchange peaks undergo exchange.

The simulations of the ^19^F spectra reproduce the minimal chemical shift changes observed for the TFSI^–^ exchange peak as a function of temperature with an exchange rate below 1 kHz, indicating a slower exchange rate than used for the Li^+^ ions. Thus, the exchange resonances for the TFSI^–^ anions are in the “slow-to-intermediate” regime relative to the NMR timescale and no coalescence between the peaks from the two subsets of ions is seen even at the highest temperature studied. Thus, the spectral simulations of the ^7^Li and ^19^F exchange processes suggest that the exchange rates of the Li^+^/TFSI^–^ ions at 295 K that give rise to the exchange peaks are approximately 1 kHz and 0.6–0.8 kHz, respectively. We also recorded spectra at a higher magnetic field strength of 16.5 T (and, thus, larger separation between in- and ex-pore peaks) to support our peak assignments, simulations with the same exchange rates also providing a good match to these spectra (see Supporting Information Section 7).

### Quantitative Analysis of Ion Adsorption by NMR

**Figure 4:**
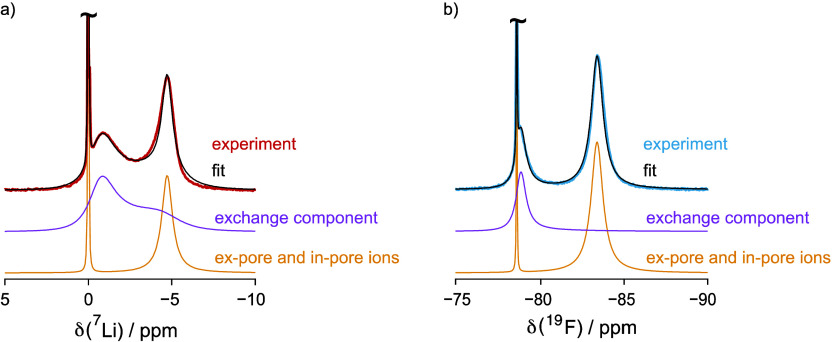
(a) ^7^Li and (b) ^19^F NMR MAS spectra of the carbon sample saturated with an electrolyte (colored in red and blue) and deconvolution into two components: an “exchange” component (colored in lilac) and “free and in-pore ions” components (colored in yellow). The exchange components for ^7^Li and ^19^F were simulated with exchange rates of 1000 and 600 Hz, respectively. The sum of the two components (in black) is overlaid with the experimental spectra.

The quantification of total adsorbed in-pore ions inside the saturated carbon sample is complicated by the fraction of in-pore ions that undergo chemical exchange with ex-pore ions. The spectra were fitted with three components: an “exchange component” and “ex-pore” and “in-pore” components, as illustrated in Figure 4. The “ex-pore” and “in-pore” signals arise from ions in the slow exchange regime and can be directly quantified. The “exchange” components were fitted by using lineshapes extracted from the two-site exchange simulation in which the frequencies of the exchanging environments (“ex-pore” and “in-pore”) were constrained, only allowing the exchange rates and the population ratios between the exchanging environments to vary (see simulation details in Supporting Information Section 2). The “in-pore” ion contribution to the exchange peak was extracted from the simulation and added to the “in-pore” peak intensity to give the total quantity of in-pore ions. The resulting analysis gives total in-pore Li^+^ and TFSI^–^ concentrations in YP-50F carbon of 1.6 ± 0.1 and 1.7 ± 0.1 mmol g^–1^, respectively. The error in the fitting mainly comes from the fact that the exchange component shown here was simulated with one discrete exchange rate and single values for the chemical shifts of the in- and ex-pore signals, but in reality, there will be both a distribution of exchange rates and chemical shifts of the in-pore peaks. The reported error is the standard deviation from fitting the exchange peaks with slightly different population ratios and exchange rates.

Finally, the ^1^H NMR spectra (shown in Figure S9a) were acquired, and again, resonances were observed from in-pore, ex-pore, and exchange peaks. These were then deconvoluted using the same method as described above to determine the molar fractions of in- and ex-pore water. Assuming that the density of water within the electrolyte remains the same in and out of the pores (i.e., the intensity of the water signal is directly proportional to the volume, independent of whether this signal comes from in- or ex-pore environments), in-pore ion concentrations of 3.1 ± 0.2 and 3.3 ± 0.1 M were calculated for the Li^+^ and TFSI^–^ ions, respectively, compared to the ex-pore ion concentrations of 0.7 ± 0.2 and 0.6 ± 0.1 M for Li^+^ and TFSI^–^, respectively (details in Supporting Information Section 8). These results show that the in-pore ion concentration is significantly higher than the ex-pore concentration, confirming that the ions prefer to be adsorbed inside carbon pores, i.e., the carbon is ionophilic in an aqueous 1 M LiTFSI electrolyte. There is little evidence for preferential sorption of Li^+^ over TFSI^–^ or vice versa at this pH (7.03) or salt concentration.

### NMR Studies of the Impact of pH on Ion Adsorption

**Figure 5:**
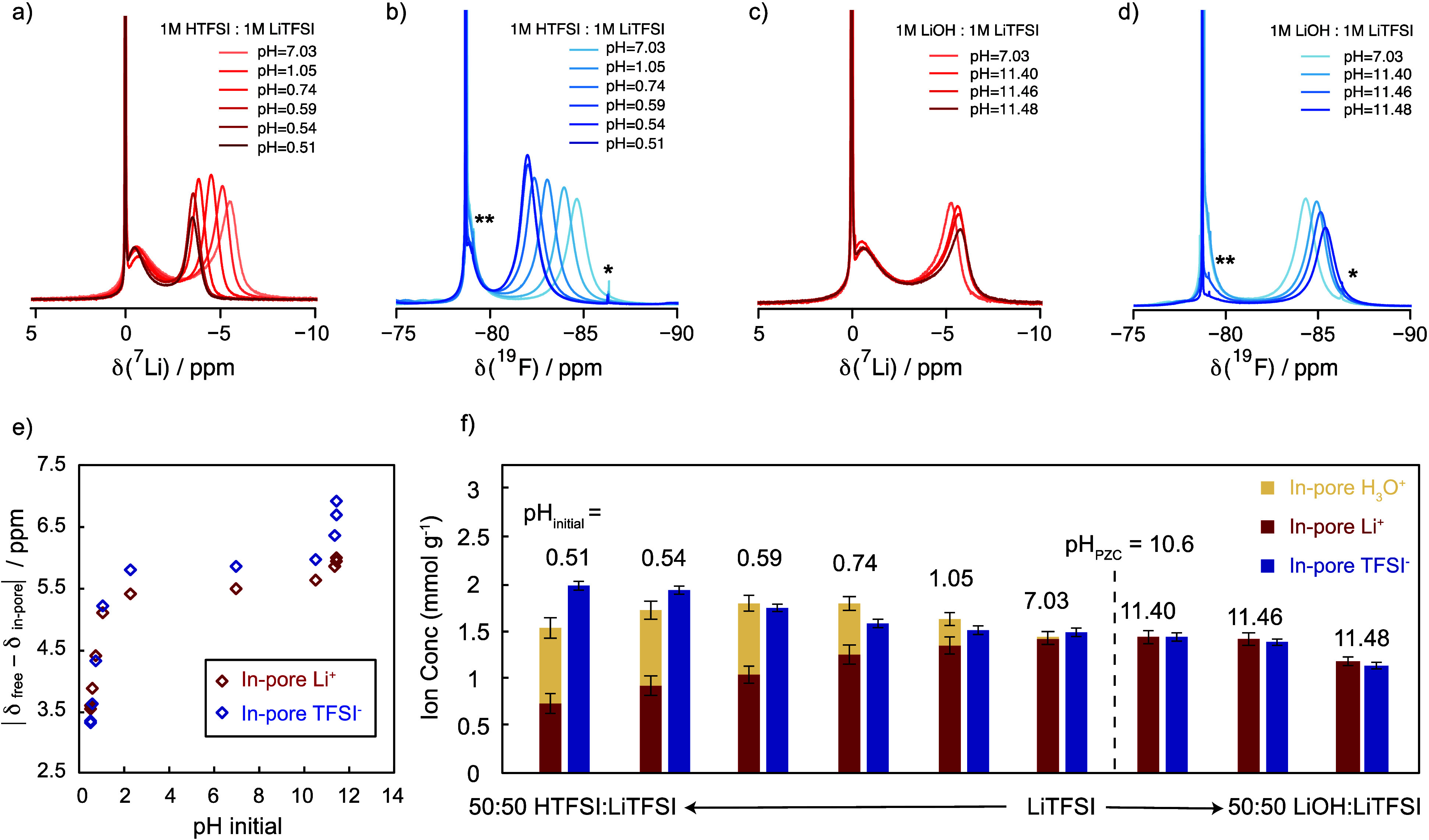
(a) ^7^Li and (b) ^19^F MAS NMR spectra of YP-50F carbon saturated with a 1 M LiTFSI aqueous electrolyte mixed with 50, 40, 30, 20, 10, and 0%, 1 M HTFSI electrolyte (v/v). The pH values of the resulting LiTFSI electrolytes were pH = 0.51, 0.54, 0.59, 0.74, 1.05, and 7.03, respectively. (c) ^7^Li and (d) ^19^F NMR spectra of YP-50F carbon fully filled with a 1 M LiTFSI electrolyte mixed with 50, 30, and 10%, 1 M LiOH electrolyte (v/v). The pH values of the resulting LiTFSI electrolytes were pH = 11.48, 11.46 and 11.40, respectively. (e) Chemical shift difference between ex-pore and in-pore ions (Δδ) as a function of pH of the LiTFSI electrolyte. (f) In-pore ion concentrations extracted from ^7^Li and ^19^F NMR spectra and pH measurements. The single asterisk (*) and double asterisks (**) in the ^19^F spectra denote the spinning sideband (MAS = 5 kHz) and a ^1^J(^13^C–F) doublet of 320 Hz, respectively. (All spectra were measured at 7.1 T, and they are normalized by the mass of the carbon sample.)

To investigate the role that adsorbed H_3_O^+^ ions play in maintaining local charge neutrality, ^7^Li and ^19^F NMR spectra were acquired for YP-50F carbon samples saturated with a range of LiTFSI–HTFSI/LiOH electrolytes with different pH values (Figure 5a–d). Across the pH range, three spectral features are clearly visible across all samples for Li^+^ and TFSI^–^ ions, corresponding to the ex-pore ions, exchanging ions, and in-pore ions. The chemical shifts of the ex-pore ions at different pH levels are constant for both ^7^Li (0 ppm) and ^19^F NMR (−78 ppm), suggesting that the chemical environments of Li^+^ and TFSI^–^ in the electrolyte are not significantly affected by pH. However, for both ^7^Li and ^19^F, significant shifts of the in-pore resonances are observed across the pH range: the more acidic the LiTFSI electrolyte is, the more the in-pore resonance shifts toward less negative ppm; the more basic the LiTFSI electrolyte is, the more the in-pore resonance shifts toward more negative ppm. The separation between in-pore and ex-pore ions (Δδ) is smaller under acidic conditions and larger under basic conditions, as summarized in Figure 5e; the similarity of this curve to the H_3_O^+^ adsorption curve in Figure 1c is striking and is discussed further in the discussion section below.

The effect of ion concentration on the NMR chemical shifts has been known in the literature. Cervini et al. have shown in their PDCs that as the concentration of the injected electrolyte was reduced from 1 to 0.05 M, the in-pore chemical shift separation (Δδ) was reduced significantly from −5.2 to −0.3 ppm.^37^ They attributed the reduced shift separation at lower electrolyte concentration to the redistribution of the in-pore ions within the porous network, with favored occupancy of larger pores at lower concentrations. While our work suggests that the increased role of hydronium and hydroxide ions at low concentrations may need to be considered, we have also examined whether the shift of in-pore resonances in response to changes in electrolyte pH seen in this current work is a consequence of variations in ion concentration. The ^19^F, ^7^Li, and ^1^H NMR spectra were recorded on the YP-50F carbon sample saturated with 1, 0.75, and 0.5 M LiTFSI aqueous electrolytes (Figure S10), with the in-pore chemical shift separation decreasing by only 0.36 ppm for ^7^Li. The lower limit of 0.5 M was chosen such that it matches the Li^+^ ion concentration of 0.5 M in the most acidic sample (50%:50% v/v of LiTFSI:HTFSI, pH = 0.51). The changes in ^7^Li shift between the 1 M and 0.5 M LiTFSI electrolyte sample (0.36 ppm) are significantly smaller than the ^7^Li shift changes observed between the neutral and the most acidic samples (1.91 ppm).

In addition to the shifts of the in-pore resonances, the intensity of the in-pore resonances also varies as a function of pH. Combining the in-pore Li^+^ and TFSI^–^ NMR quantifications (performed as described in Quantitative Analysis of Ion Adsorption by NMR) with the in-pore H_3_O^+^ pH quantifications obtained with the 1 M LiTFSI electrolytes (Figure 1d) provides a full picture of the different charged species adsorbed inside the carbon pores at different pH levels (Figure 5f). The quantification of ^7^Li NMR spectra again assumes that the exchange of Li^+^ ions is in the fast exchange regime, while ^19^F NMR spectra are assumed to be in the slow-intermediate regime. The quantification errors for Li^+^ and TFSI^–^ uptake are again estimated based on the standard deviation from different fits of the exchange peaks. The real error of the ion quantification is likely larger and systematic since there may be errors in the determination of the relative contributions of in- and ex-pore environments to the Li^+^ exchange peak, as discussed in Quantitative Analysis of Ion Adsorption by NMR. These errors will be the largest in the highly acidic samples where the separation between ex-pore and in-pore peaks is the smallest. Despite these errors, the trend that the H_3_O^+^ ions in acidic electrolytes participate in maintaining the charge neutrality is robust. Furthermore, there is a noticeable and steady increase in total ion uptake from basic to acidic electrolytes. Only at the lowest pH values (pH < 0.59) does the cation uptake appear to decrease (as discussed further below).

### Electrochemical Measurements of the Impact of pH on Capacitances

**Figure 6:**
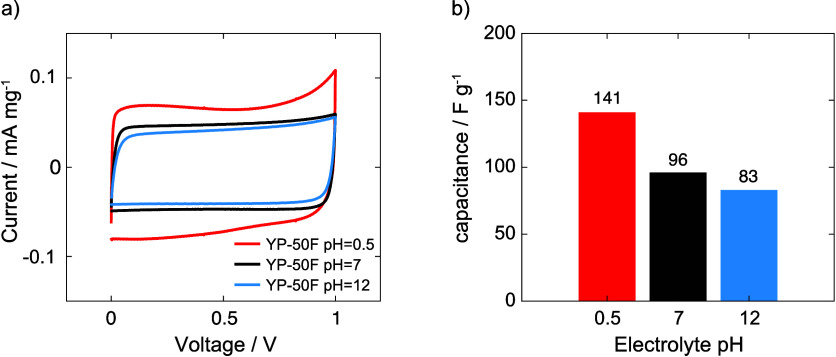
(a) Cyclic voltammograms of YP-50F carbon in acidic (50:50 mixture of 1 M HTFSI and 1 M LiTFSI, pH = 0.51), neutral (1 M LiTFSI, pH = 7.03), and basic (50:50 mixture of 1 M LiOH and 1 M LiTFSI, pH = 11.48) electrolytes between 0 and 1 V at a scan rate of 0.5 mV/s. (b) Capacitances measured from galvanostatic charge–discharge experiments of YP-50F carbon in acidic, neutral, and basic LiTFSI electrolytes at a constant charge/discharge current of 0.2 mA/g.

So far, the ion adsorption in carbon has been studied in the absence of any applied potential. To further explore the effect of pH on the ion adsorption and subsequently on the charge stored in a supercapacitor device, symmetric two-electrode YP-50F carbon supercapacitors were assembled and charged from 0 to 1 V with three 1 M LiTFSI aqueous electrolytes across the pH range (pH = 0.51, 7.03, and 11.48). The resulting cyclic voltammograms exhibit near-rectangular shapes, indicating that ionic charges are predominantly stored as electric double layer capacitance in YP-50F carbon at all pH values. The capacitance of YP-50F in different pH electrolytes was further assessed through galvanostatic charge and discharge experiments, as summarized in Figure 6b. A maximum capacitance of 141 F/g was attained in the acidic LiTFSI electrolyte (pH = 0.51), followed by capacitances of 96 and 83 F/g in the neutral LiTFSI electrolyte (pH = 7.03) and basic LiTFSI electrolyte (pH = 11.48), respectively. The increased capacitance in the acidic electrolyte correlates with the intrinsically higher in-pore ion concentration seen in the NMR titration experiments (Figure 5f), providing more ions in the pores for charge storage. Further studies on the ion adsorption during charging via operando NMR spectroscopy are in progress to obtain a more detailed mechanistic understanding of charge storage in aqueous supercapacitors and to relate deviations in the rectangular shapes to, for example, (de)protonation of functional groups with the state of charge.^9^

## Discussion

### Interfacial Charge Storage at Different pH Levels

Our study shows the importance of quantifying H_3_O^+^ adsorption and determining the nature of the acid/basic groups when quantifying the ionic species adsorbed in porous carbons. With the presence of both internal and external surface functional groups, the carbon surface will become charged once in contact with an aqueous electrolyte as dictated by the point of zero charge (10.6 ± 0.2) in the 1 M LiTFSI electrolyte. To a first approximation, the basic functional groups will dominate the pH dependence of the adsorption process, with approximately five times more basic groups than acidic groups (Table 1). Even the acidic groups are dominated by very weak phenol-type species, which will only be fully deprotonated under the more basic conditions.

**Figure 7:**
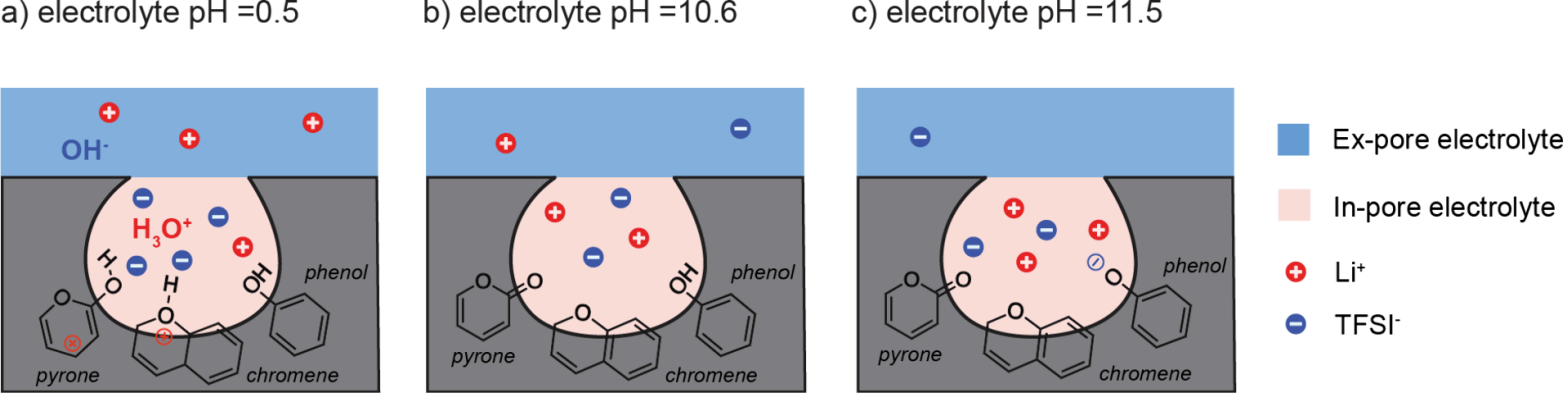
Illustrations of the ion adsorption inside carbon pores with the pyrone, chromene, and phenol functional groups at the following conditions: (a) electrolyte pH_initial_ = 0.5 where pyrones and chromenes are protonated (the protonated carboxylic and lactone groups are not shown in the figure), (b) electrolyte pH_initial_ = 10.6 where the overall charge of the surface is neutral, and (c) electrolyte pH_initial_ = 11.5 where the phenolic and other acidic groups are deprotonated.

For an electrolyte with pH ≪ PZC, the carbon surface will become positively charged due to the protonation of the excess basic functional groups. As the pH of the initial electrolyte approaches pH = 0.5, the amount of adsorbed H_3_O^+^ approaches 0.8 mmol g^–1^ under the conditions used in our NMR experiments. Even at this point, not all basic groups are protonated, as the Boehm titration with an excess of 0.05 M HCl indicates that there is a total of 1.2 mmol g^–1^ basic groups. While the H_3_O^+^ ions bound to the basic groups are dynamic and in rapid equilibrium with the water in the aqueous salt electrolyte, they are chemically bound to the carbon surface and the resulting changes to the carbon surface will have an impact on the double layer formation with charged salt ions (Li^+^ and TFSI^–^). The simplistic figure shown in Figure 1b needs to be modified accordingly (Figure 7): under acidic conditions (initial electrolyte pH = 0.51, Figure 7a), the surface carries a net positive charge of approximately 0.8 mmol g^–1^ arising from the protonation of both acidic and basic functional groups (chromene and pyrone groups) on the carbon internal and external surfaces. Under excess HCl (pH zero) and without Li^+^ ions competing, this surface charge approaches 1.2 mmol g^–1^, attributed to the further deprotonation of phenols; the experiments suggest that Li^+^ ions compete with H_3_O^+^ ions at higher Li^+^ concentrations. At pH = 10.6 ± 0.2, the basic groups are no longer protonated and the carbon surface is uncharged (Figure 7b). For pH ≫ PZC, the phenols start to be deprotonated and the carbon surface is now negatively charged (Figure 7c). The surface reaches a net negative charge of 0.2 mmol g^–1^ in excess 0.05 M NaOH (pH 13) in the Boehm titration (Table 1).

With this view of the continually modifying carbon (internal and external) surface, we now reinterpret the NMR quantification results presented in Figure 5f. Only at a pH of 10.6 do we expect the surface charge to be zero and the number of salt ions in the double layer to be equivalent (Figure 7b), which is indeed what is observed experimentally at pH 11.40 (Figure 5f), close enough to the PZC so that the initial pH and final pH are almost the same (i.e., there is no net sorption of a proton or hydroxide ion). At this point, we observe both local and bulk charge neutrality. As we reduce the pH, protons from the H_3_O^+^ ions will be increasingly chemisorbed (Figure 7a). When an electrolyte with an initial pH of 1.05 is used, the final “ex-pore” electrolyte has a pH of 7.05, indicating that 0.3 mmol g^–1^ H_3_O^+^ ions have been adsorbed. Since the pH 7.05 “ex-pore” electrolyte is in equilibrium with the carbon, many but not all of the basic groups will be protonated, as determined by the p*K*_b_ of the basic groups. As a result of the partially positive charge on the carbon surface, we suggest that this favors a higher uptake by TFSI^–^ over Li^+^ to partially compensate for the carbon’s positive surface charge (Figure 5f). At a pH of 0.74, where the ex-pore electrolyte now has a pH of less than 4.0, the uptake of protons has increased to 0.6 mmol g^–1^, with the TFSI^–^ uptake increasing further (Figure 5f). At this point, the total cation uptake is noticeably higher than the TFSI^–^ uptake, but this is because the cation uptake originates from both the Li^+^ adsorption in the pores and chemisorbed protons (i.e., those tightly bound to the basic functional groups). There are very few hydronium ions (protons) (10^–4^ M) in the electrolyte at pH 4. The situation changes for the electrolyte with an initial pH of 0.51, where the ex-pore electrolyte approaches pH 1.33 on equilibration. Now, the H_3_O^+^ concentration in the ex-pore electrolyte is around 0.1 mmol g^–1^. The TFSI^–^ concentration in the pores approaches 2 mmol g^–1^, an excess of 1.3 mmol g^–1^ over the measured Li^+^ concentration (pH 0.51 in Figure 5f). A chemisorbed proton concentration of 0.8 mmol g^–1^ is observed, and we are now “missing” approximately 0.5 (±0.2) mmol g^–1^ of positive charge via NMR and pH studies (Figure 5f), with the carbon appearing to be negatively charged, via this analysis. While we acknowledge a large error associated with Li^+^ exchange peak quantification, we do not believe this to be the only source of this difference. Instead, we ascribe some of this difference to additional H_3_O^+^ ions present in the pores that have not been quantified via the pH measurements.

To illustrate, if we assume that the carbon surface contains no functional groups that can be (de)protonated, then the pH remains unchanged on adding the electrolyte, assuming no preferential uptake of Li^+^ or TFSI^–^. If we assume the pH inside the pores to be equal to that outside, then with a pH of 1 and the pore volume of 0.7 cm^3^ g^–1^ measured for YP-50F,^16^ the 1 g of carbon used in this experiment should contain approximately 0.07 mmol of H_3_O^+^ in its pores. At a pH of 0.5, the H_3_O^+^ concentration should increase to 0.22 mmol g^–1^ inside the pores. These hydronium ions in the pores are not accounted for in our pH measurements—we only account for the ions that are bound to the surface and change the pH of the electrolyte (ex-pore) solution. While this is still not enough to account for the difference of 0.5 mmol g^–1^, we are closer to approaching overall charge neutrality. Finally, we need to consider how measurements are performed. To quantify total H_3_O^+^ uptake, we took a carbon that has been washed multiple times with approximately pH 7 deionized water, followed by drying. Before drying at least, our PZC measurements confirm that this carbon must be partially protonated because the washing solutions were at pH lower than the PZC. However, after drying, this carbon still sorbs H_3_O^+^ ions. This uptake can be estimated from Figure 1c, i.e., the change of a pH_initial_ from 7 to 10.6, and it is this H_3_O^+^ content that has been accounted for in our analysis of charge balance. Thus, errors occur because the initial degree of protonation may not be accounted for. Finally, the total basic group concentration determined with 0.05 M HCl is 1.2 mmol g^–1^, in contrast to the 0.8 mmol g^–1^ determined with 1 M LiTFSI, indicating that some functional groups are still not fully protonated and proton uptake is sensitive to other factors including total Li^+^ ion concentration.

By contrast, for an initial electrolyte pH of 11.48, the pH values of the initial (pH_initial_) and ex-pore electrolytes (pH_final_) are essentially the same (Figure 1c). At this point, the carbon will be slightly negatively charged, with deprotonation of the various acidic groups being affected by their p*K*_a_ (Figure 7c): at this pH, the acidic groups should be deprotonated, but since they are present in very small quantities, the carbon negative charge will be negligible. This is consistent with the barely detectable higher uptake of Li^+^ over TFSI^–^ at pH = 11.48 (Figure 5f).

The total ion uptake concentration increases noticeably on going from basic to acidic electrolytes, with TFSI^–^ increasing from 1.2 to 2.0 mmol g^–1^ at the most acidic pH studied here. The overall measured charge carrier concentration including the contribution from H_3_O^+^ ions inside the pores increases from 2.5 mmol g^–1^ in basic electrolytes to 3.5 mmol g^–1^ in acidic electrolytes, indicating increased ionophilicity. We ascribe the increased ionophilicity to the protonation of the basic groups, which then in turn results in an increased uptake of the charge-compensating TFSI^–^ groups, with the total uptake of H_3_O^+^ (0.8–1.2 mmol g^–1^, depending on pH and salt concentration) being of the same order as the increased TFSI^–^ uptake. The ionophilic nature of carbon in acidic electrolytes has implications on the charge storage mechanisms in aqueous electrolyte-based supercapacitor devices and is consistent with the higher capacitance measured for YP-50F in the acidic LiTFSI electrolyte of 141 F g^–1^ compared to the capacitances of 96 and 83 F g^–1^ in neutral and basic LiTFSI electrolytes, respectively (Figure 6), with these values being similar to the literature value of 85 F g^–1^ for YP-50F in 1 M KOH.^38,39^ Indeed, a recent theoretical paper has highlighted the additional capacitance that results from the protonation of basic groups on graphene sheets.^40^

The clear ionophilic nature of YP-50F is in contrast to earlier work by Cervini et al. on their PDCs; this system is ionophobic with respect to hydrated Li^+^ and Na^+^ ions in aqueous LiCl and NaCl electrolytes, leading to a lower in-pore than ex-pore ion concentration.^37^ We suggest that the difference between PDCs and YP-50F lies in the nature and quantity of (internal) surface groups on PDCs, motivating further studies of the functional groups in this class of carbon. Previous studies have shown that ionophobic pores can accelerate the charging process in non-aqueous-electrolyte supercapacitors by avoiding the slow diffusion caused by overfilling.^41^ However, ionophobic carbons will also show lower capacity.^42,43^

### pH Dependence of the In-Pore Chemical Shift

As the electrolyte becomes more acidic, a decrease in the chemical shift separation, Δδ, between ex-pore electrolyte ions and in-pore ions is observed; furthermore, a noticeable increase in Δδ occurs once the carbon becomes negatively charged for both anion and cation signals (Figure 5e). Given that Δδ originates from local magnetic fields due to the circulation of the delocalized π-electrons in the aromatic rings of the internal carbon surfaces,^36^ a decrease in the chemical shift separation suggests a reduced carbon ring current effect. Previous studies have shown that the magnitude of the shifts depends on the pore sizes, the average distance between the nucleus being measured and the carbon surface, and the degree of ordering in the aromatic regions in the carbons.^36^ While the pore sizes will not be affected by pH, the other two factors may be. Furthermore, it is intriguing that the trend in Δδ as a function of electrolyte pH (Figure 5e) mirrors the shape of the curve in the H_3_O^+^uptake study (Figure 1c), which further suggests that there is an underlying correlation between the amount of H_3_O^+^/OH^–^ adsorption and the average ring currents seen by the adsorbed ions.

**Scheme 1:**
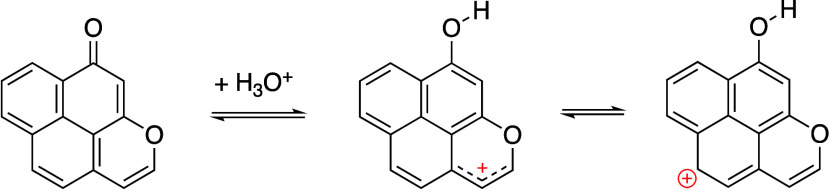
Protonation of Pyrone Stabilized through Delocalization into the Carbon Aromatic Ring Structure

One potential explanation that we now explore is that the protonation of the carbon surface groups for samples with electrolyte pH ≪ PZC causes an inductive effect that withdraws electron density from the delocalized aromatic carbon rings, weakening the carbon ring current effect and accounting for the observed chemical shift behavior.^44^Scheme 1 shows the protonation of a pyrone considered by Boehm and Suarez et al. in their DFT studies.^45^ Protonation of the carbonyl group can be stabilized by the formation of a phenolic group, with the associated aromaticity of the phenolic ring, but this requires the movement of the resulting positive charge into the adjacent heterocyclic ring. Since the heterocyclic ring is directly bound to the aromatic ring system (see, for example, the larger fragment considered by the same DFT study), the charge can further be delocalized into the aromatic ring structure (Scheme 1). To test this hypothesis, NICS calculations have been performed for a variety of heterosubstituted benzenes, including phenol and pyrone. However, the NICS changes near their protonated and deprotonated states do not follow a simple trend with the extent of protonation (see Supporting Information Section 10), suggesting that this is not the source of the change in Δδ values.

As discussed above, the explanation that Δδ tracks overall ion concentration as observed by Cervini et al. for their system^37^ is not sufficient to account for the larger changes in our system. Their system is ionophobic, and they observe smaller shifts at lower ion concentrations. We observe much larger shifts at pH in a regime where fewer ions are adsorbed.

The simplest explanation is that at low pH, the TFSI^–^ ions spend more time, on average, near the protonated functional groups and, thus away from the more aromatic, more graphene-like regions, resulting in smaller NICS values. While the Li^+^ ions, on average, are near these ions to maintain charge neutrality, the effect is more pronounced for the TFSI^–^ ions, hence, their smaller Δδ values. When the functional groups are deprotonated, the TFSI^–^ ions are now repelled and are, on average, closer to the more aromatic regions with fewer defects within the carbon pores (now resulting in larger Δδ values). There are fewer ions sorbed at these pH values, consistent with the exclusion of ions from regions of carbon rich in defects. The Li^+^ ions follow the TFSI^–^ ions to maintain charge balance, but some also remain near the negatively charged deprotonated functional groups to compensate for the charges, and hence, a much smaller change in Δδ is seen for ^7^Li (0.5 vs 1.1 ppm for ^7^Li and ^19^F, respectively).

## Conclusions

We have presented a systematic study of a LiTFSI aqueous electrolyte adsorbed in a (YP-50F) porous carbon by NMR spectroscopy as a function of pH. The ^7^Li and ^19^F NMR spectra reveal that the adsorbed ions are highly dynamic, constantly diffusing in and out of carbon pores. In addition to the observation of signals from ions outside (ex-pore electrolyte ions) and inside (in-pore electrolyte ions) the pores, as seen previously in organic electrolytes, we also observed an additional peak corresponding to the ions undergoing rapid exchange between ex-pore and in-pore environments, with a shift in between the in- and ex-pore peaks. As a result, any quantification of the total sorbed ion concentration needs to account for this “exchange” peak; we have achieved this in this study through simulations involving a simple two-site exchange model. In addition to quantifying the adsorbed electrolyte ions, we have also measured the H_3_O^+^ uptake through pH measurements, yielding a H_3_O^+^ adsorption capacity of 0.8 mmol g^–1^ at low pH. This H_3_O^+^ uptake is attributed to the surface basicity of the inner pore surfaces of YP-50F carbon, as characterized through the XPS measurements and quantified via Boehm titrations. The results demonstrate that (de)protonation of the internal and external carbon surfaces and the concentration of hydronium/hydroxide ions inside and outside the pores need to be taken into account when considering electroneutrality in these systems. Bringing the quantification of adsorbed H_3_O^+^ by pH measurements together with the quantification of adsorbed Li^+^ and TFSI^–^ ions by NMR spectroscopy, we find that the total number of ions (Li^+^, TFSI^–^, and H_3_O^+^) adsorbed in the pores increases under acidic conditions, indicating that the carbon becomes more ionophilic in acidic electrolytes. The increased ion uptake at low pH then correlates directly with increased capacitance, with the work clearly showing that performance is directly affected by the presence of defects.

In addition to the changes in the in-pore ion population, the ^7^Li and ^19^F chemical shift separation between the ex-pore and in-pore ion resonances is sensitive to the nature and degree of protonation of the oxygen-containing functional groups. The shift separation decreases under acidic conditions and increases at high pH (particularly for the ^19^F signals from TFSI^–^ ions), with the shift showing a strong correlation with the total H_3_O^+^ ion uptake. We ascribe this change to redistributions of ions within the carbon pores as a result of the degree of protonation of the functional groups—at high pH, the negatively charged functional groups drive the negatively ions into the more defect-poor and more highly aromatic porous regions, with the ions experiencing larger ring currents (and associated greater NICS). At low pH, the TFSI^–^ ions are now attracted by the protonated functional groups. These results have important consequences for understanding ion uptake in carbons in other aqueous systems and devices, as the methodology can also be applied to develop structure–property relations in the materials used for water desalination, electrocatalysis, and carbon capture. Our new approach provides clear guidelines on how to optimize materials for ion uptake at different pH values, which will help guide future materials discovery in this general area.

## Supporting Information Available

Additional characterizations, NMR results, detailed exchange model, and DFT calculations (PDF)
