# Supplementary material for: Understanding Sorption of Aqueous Electrolytes in Porous Carbon by NMR Spectroscopy
Source: J Am Chem Soc. 2024 Apr 1;146(14):9897–910. doi: 10.1021/jacs.3c14807 (PMC11009947; doi:10.1021/jacs.3c14807)
Supplement: Supplementary file 1 — ja3c14807_si_001.pdf [file ja3c14807_si_001.pdf]

Understanding Sorption of Aqueous Electrolytes in Porous Carbon by NMR spectroscopy

Dongxun Lyu [a], Katharina Märker [a,b], Yuning Zhou [a], Evan Wenbo Zhao [a,c], Anna B. Gunnarsdóttir [a,d], Samuel P. Niblett [a], Alexander C. Forse [a], Clare P. Grey [a*]

[a] Yusuf Hamied Department of Chemistry, University of Cambridge, Cambridge, CB2 1EW, UK
[b] Present address: Université Grenoble Alpes, CEA, IRIG, MEM, Grenoble 38000, France
[c] Present address: Magnetic Resonance Research Center, Institute for Molecules and Materials, Radboud University, Nijmegen, 6525 AJ, NL
[d] Present address: Faculty of Industrial Engineering, Mechanical Engineering and Computer Science, University of Iceland, 107 Reykjavík, Iceland
* Email: cpg27@cam.ac.uk

1) Effect of salt concentration and electrolyte ion species on PZC determination....................2
2) XPS of YP-50F carbon film...3
3) N_2 gas sorption analysis of YP-50F activated carbon 4
4) The two-site exchange model 5
5) In-pore ion concentration at variable temperatures...6
6) ^{7}Li and ^{19}F NMR exchange spectroscopy (EXSY) 7
7) Magnetic field-dependence of the ^{7}Li, ^{19}F NMR spectra...10
8) Determination of in-pore electrolyte concentration..11
9) NMR concentration experiments 12
10) NICS calculations of phenol- and pyrone- functionalised carbon coronene rings............13
References 15

1) Effect of salt concentration and electrolyte ion species on PZC determination

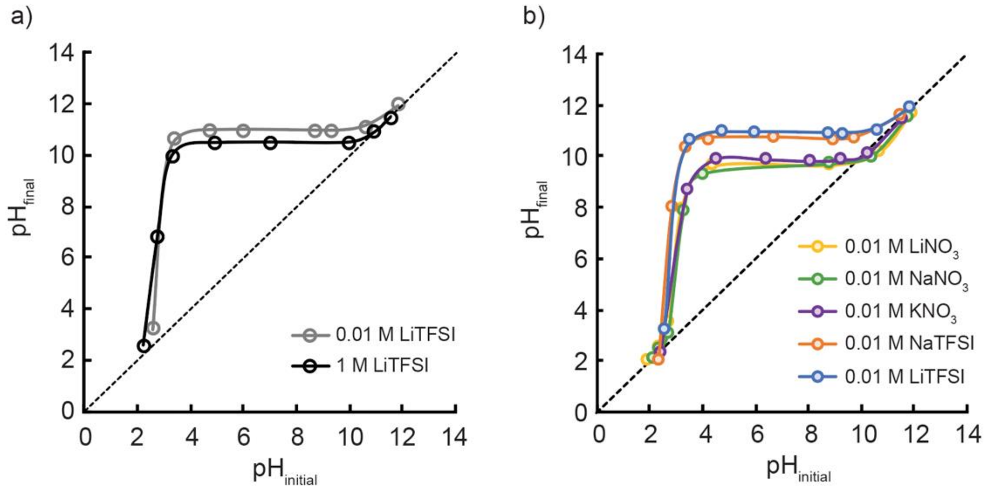

Figure S1. *PZC measurements of YP-50F in a) 1 M and 0.01 M LiTFSI electrolytes, and b) 0.01 M LiNO$_3$, NaNO$_3$, KNO$_3$, NaTFSI and LiTFSI electrolytes.*

The PZC of YP-50F was studied in 0.01 M LiTFSI electrolyte to compare with the PZC measurements discussed in the main text, performed in 1 M LiTFSI electrolyte, so as to study the effect of salt concentration on H_3O^+ adsorption. As shown in Figure S1a, a higher PZC for YP-50F in 0.01 M LiTFSI electrolyte of 11.0 was determined, as compared to 10.6 for a 1 M LiTFSI electrolyte. The higher H_3O^+ uptake in 0.01 M LiTFSI is attributed to the lower concentration of Li^+ ions (0.01 M) and the resulting reduction in competition with H_3O^+ ion for binding to the basic functional groups within the pores.

The effect of different cations was also investigated by measuring the PZC of YP-50F in 0.01 M $LiNO_3$, $NaNO_3$, KNO_3, as shown in Figure S1b. The PZC of all three nitrate-based electrolytes does not vary significantly, with a value of about 9.8, which suggests that the cation species have little impact on the H_3O^+ uptake at this low salt (cation) concentration, where binding by the H_3O^+ governs the PZC. However, when the anion species is changed from $NO_3^–$ to $TFSI^–$, the PZC of both the Li^+- and Na^+-based electrolyte shows a noticeable increase from 9.6 to close to 11.0 in both 0.1 M NaTFSI and LiTFSI electrolytes, as shown in Figure S1b. Both HNO_3 and HTFSI are strong acids, and fully dissociated in the bulk electrolyte, but with HTSI being the stronger (super-) acid. The higher acidity of HTFSI will allow even weaker basic groups to be protonated, likely increasing proton uptake and thus the PZC. Under basic conditions, the competition between Li^+ - $OH^–$, Li^+ - $TFSI^–$ and Li^+ - $NO_3^–$ ion-pair interactions, vs. Li^+ - phenoxide/lactone/carboxylate (surface) interactions will influence the PZC, motivating a wider study of the effect of salt concentration and nature of the salt on the PZC.

2) XPS of YP-50F carbon film

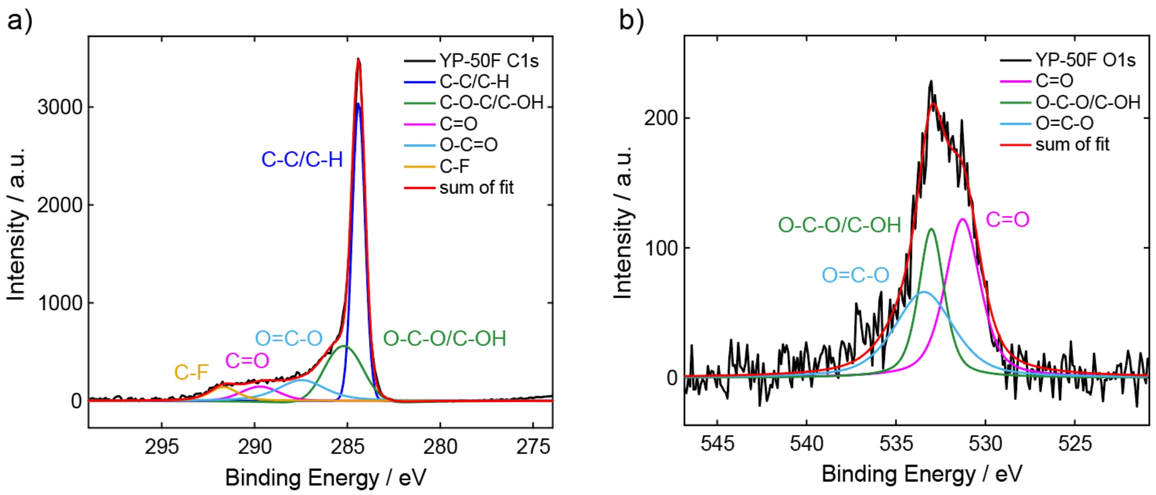

Figure S2*. the XPS spectra of a) C 1s and b) O 1s region of YP-50F carbon film.*

3) N_2 gas sorption analysis of YP-50F activated carbon

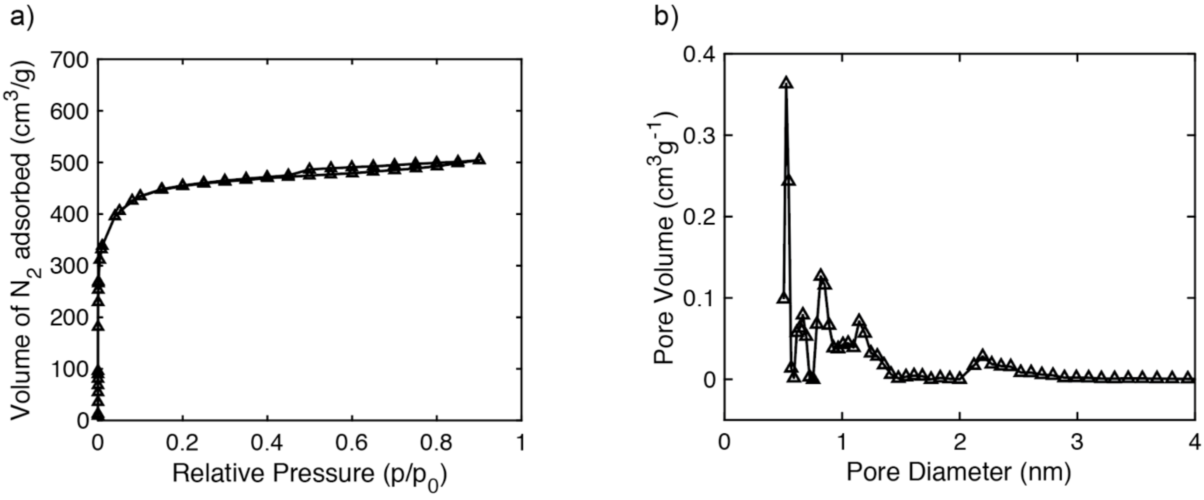

Figure S3. *Results obtained from the N_2 gas sorption analysis: a) the N_2 physisorption isotherm for YP-50F carbon powder. b) the pore size distribution plot of YP-50F carbon calculated by quench solid density functional theory based on the slit-pore model.*[1] *The pore size distribution of YP-50F carbon shows that the majority of the pores are under 2 nm.*

4) The two-site exchange model

The expected chemical exchange line shape (L_{ex}) is calculated using the formula derived by Norris:[2,3]

$$L_{ex} = \frac{-i\sum_n F_n}{1+k_{ex}\sum_n F_n} \cdot \frac{1}{\nu_L(X)\cdot 10^{-6}} \qquad \textit{Equation S1}$$

with k_{ex} being the exchange rate in Hz and $\nu_L(X)$ the Larmor frequency of the respective nucleus (^{19}F or ^{7}Li) in Hz, used to convert the spectrum to units of ppm. $\sum_n F_n$ represents a sum over the free and in-pore exchanging environments with

$$F_n(\nu) = \frac{P_n}{i(\nu - \nu_n) - \nu_{1/2,no-exch} - k_{ex}} \qquad \textit{Equation S2}$$

Here, ν_n is resonance frequency of the respective environment in Hz. $\nu_{1/2,no-exch}$ is the full width at half maximum (in Hz) of the signal of the environment without exchange. P_n is the probability of the respective environment i.e. the population of free and in-pore environments. The ratio of $P_{ex-pore}$: $P_{in-pore}$ was optimised until the simulated exchange peak resembles the experimental spectra. The resonance frequency and line width information about the free and the adsorbed in-pore environments at zero exchange rate were measured based on the ^{7}Li and ^{19}F spectra at 255 K. Due to the limitation of the cooling unit, the sample could not be cooled down further to a lower temperature. At 255 K, the exchange peak disappeared, suggesting the exchange rate is in the slow regime where coalescence cannot occur. The full width at half maximum of in-pore signal at 255 K was 210 Hz for ^{7}Li and 1140 Hz for ^{19}F.

5) In-pore ion concentration at variable temperatures

The in-pore ion population was calculated as a function of temperature, taking into account both the intensities of the in-pore signal and the in-pore component in the exchange peak from 260 K to 321 K, as shown in Figure S4. The results show that the ion concentration in the pores remains essentially constant until the lowest temperature, where freezing of the water in the electrolyte is thought to have occurred.

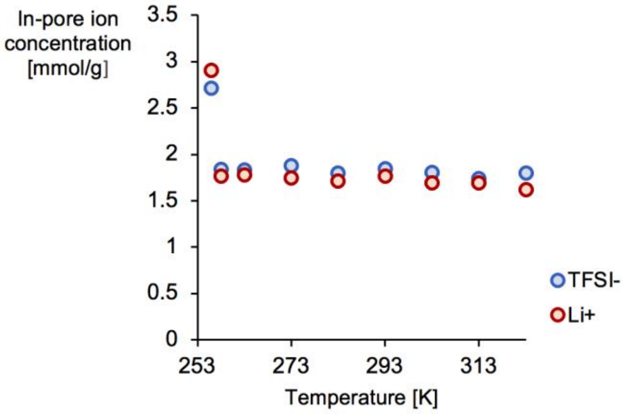

***Figure S4**. The in-pore ion concentration of Li^+ and $TFSI^-$ across the temperature range.*

In addition, the changes of lineshape in ^{7}Li spectra with temperature were fully reversible. ^{7}Li NMR spectra of the carbon sample was first recorded at 293 K and then the sample was heated to 323 K; this was followed by a gradual decrease of temperature until 263 K. Figure S5a shows the ^{7}Li NMR spectra plotted as a function of time. Figure S5b is the overlap of the spectra taken initially at 293 K (labelled as ‘293 K 1st’ and coloured in black) and the spectra taken during cooling at 293 K (labelled as ‘293 K 2nd’ and coloured in red). The fully reversible changes in the peak height and lineshape indicate that no irreversible sorption processes or changes in wetting were seen.

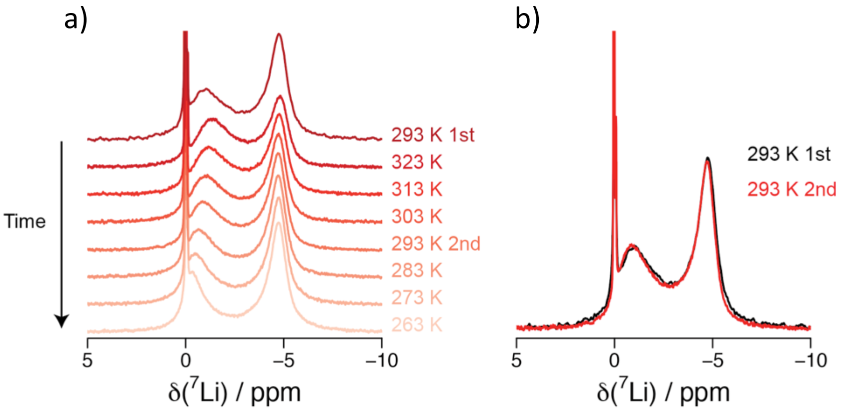

***Figure S5**. a) ^{7}Li NMR spectra as a function of time. b) ^{7}Li NMR spectra at 293 K before and after heating.*

6) ^{7}Li and ^{19}F NMR exchange spectroscopy (EXSY)

To confirm the ion exchange processes, ^{7}Li and ^{19}F EXSY experiments were recorded using a Bruker Avance III spectrometer operating at a magnetic field strength of 16.5 T (700 MHz ^{1}H Larmor frequency) using a Bruker 4mm HXY triple-resonance probe. All experiments were performed at a spinning rate of 5 kHz. For each exchange experiment, 16 transients (for both ^{7}Li and ^{19}F) were coadded for each of the 256 increments in the indirect dimension. A relatively shorter recycle delay of 1 s (compared to 60s and 30s used for both ^{7}Li and ^{19}F, respectively, in the quantitative 1D measurements) was used in both ^{7}Li and ^{19}F EXSY experiments to obtain multiple two-dimensional spectra within a reasonable time. For each experiment, 6 μL of 1 M LiTFSI electrolyte was added to 2 mg of YP-50F carbon powder inside the 4 mm NMR rotor.

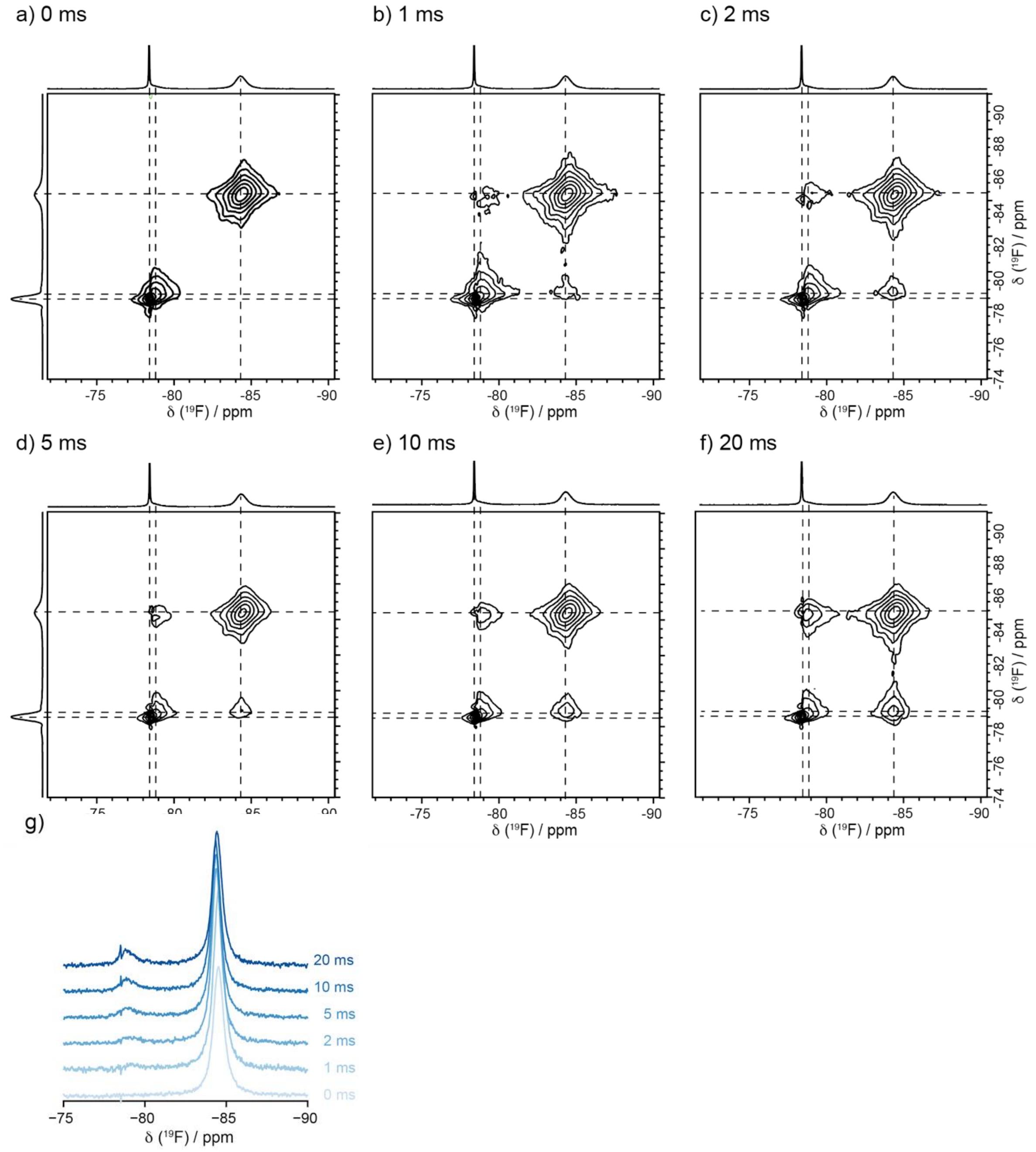

Figure S6. *^{19}F EXSY NMR spectra of the YP-50F carbon soaked with 1 M LiTFSI aqueous electrolyte at a spinning rate of 5 kHz, recorded with mixing time of a) 0, b) 1, c) 2, d) 5, e) 10 and f) 20 ms. g) slices extracted from the ^{19}F EXSY experiments as a function of mixing time.*

Representative ^{19}F EXSY spectra for YP-50F carbon powder soaked with 1 M LiTFSI aqueous electrolyte are shown in Figure S6 as a function of mixing time. The cross peaks between in-pore and exchange peaks start to emerge at a mixing time of 1 ms (Figure S6b), indicating the $TFSI^-$ ion exchange process. At a much longer mixing time of 20 ms (Figure S6f), a weak cross peak is observed connecting the in-pore and free electrolyte peak, as noticed in the extracted slices in Figure S6g.

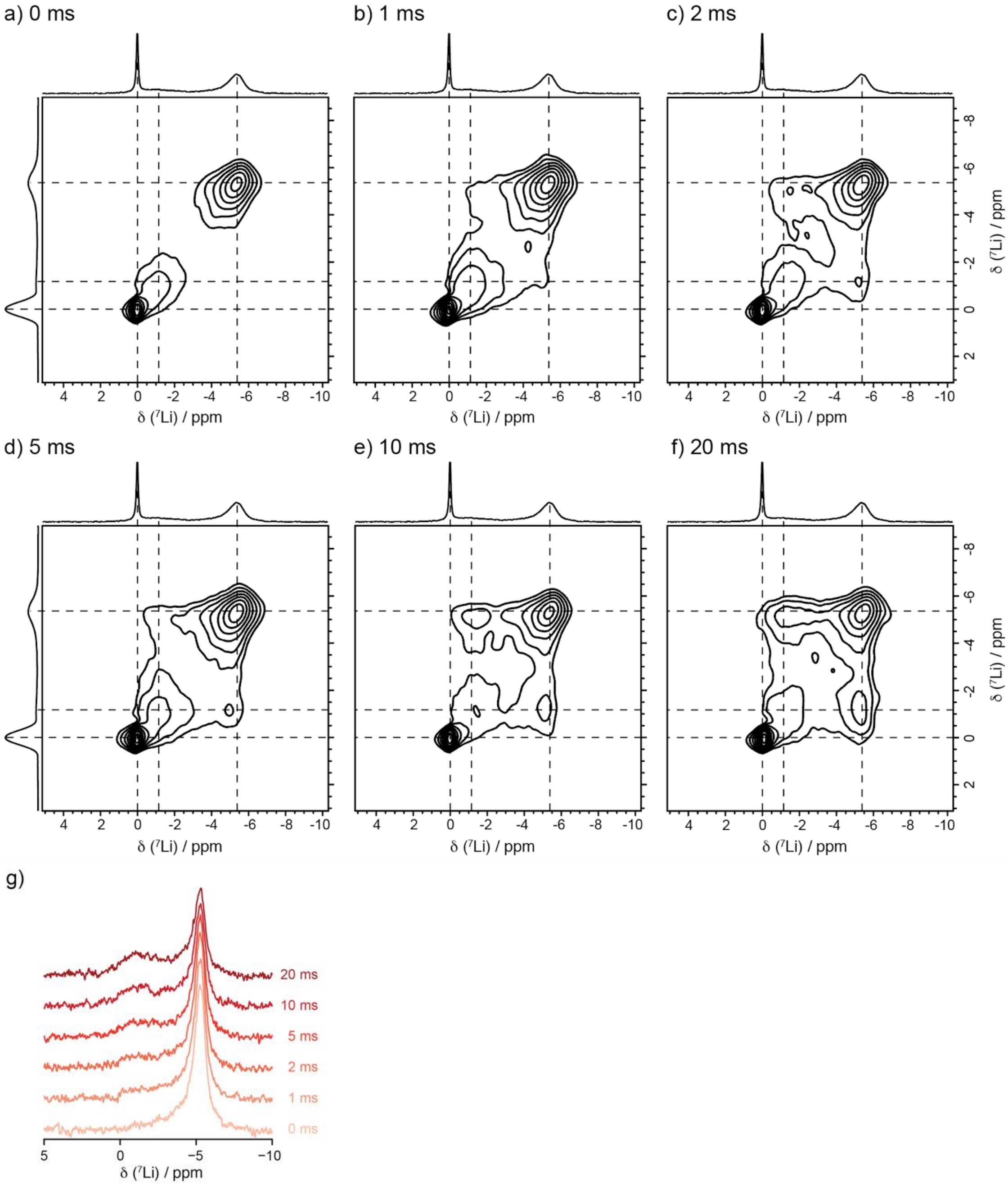

Figure S7. *^7Li EXSY NMR spectra of the YP-50F carbon soaked with 1 M LiTFSI aqueous electrolyte at a spinning rate of 5 kHz, recorded with mixing time of a) 0, b) 1, c) 2, d) 5, e) 10 and f) 20 ms. g) slices extracted from the ^7Li EXSY experiments as a function of mixing time.*

Similar cross peaks are observed for ^7Li, as shown in Figure S7. At zero mixing time (Figure S7a), only resonances on the autocorrelation diagonal are observed at in-pore (0 ppm), exchange (-1.2 ppm), and free (-5.2 ppm). Using a longer mixing time of between 1 to 20 ms (Figure S7b-g), significant cross peak intensity is observed correlating the in-pore and exchange resonances, indicating the exchange of

Li^+ between these environments. The absence of ^7Li cross peak between in-pore and free electrolyte is likely due to the short recycle delay of 1 s used in the 2D ^7Li EXSY. The longitudinal relaxation of ^7Li free electrolyte signal is around 12 s, and thus this signal is suppressed in the 2D EXSY experiment (acquired with 1 s recycle delay), hindering further interpretation of the ^7Li ion exchange process between in-pore and free electrolyte environments.

7) Magnetic field-dependence of the ^{7}Li, ^{19}F NMR spectra

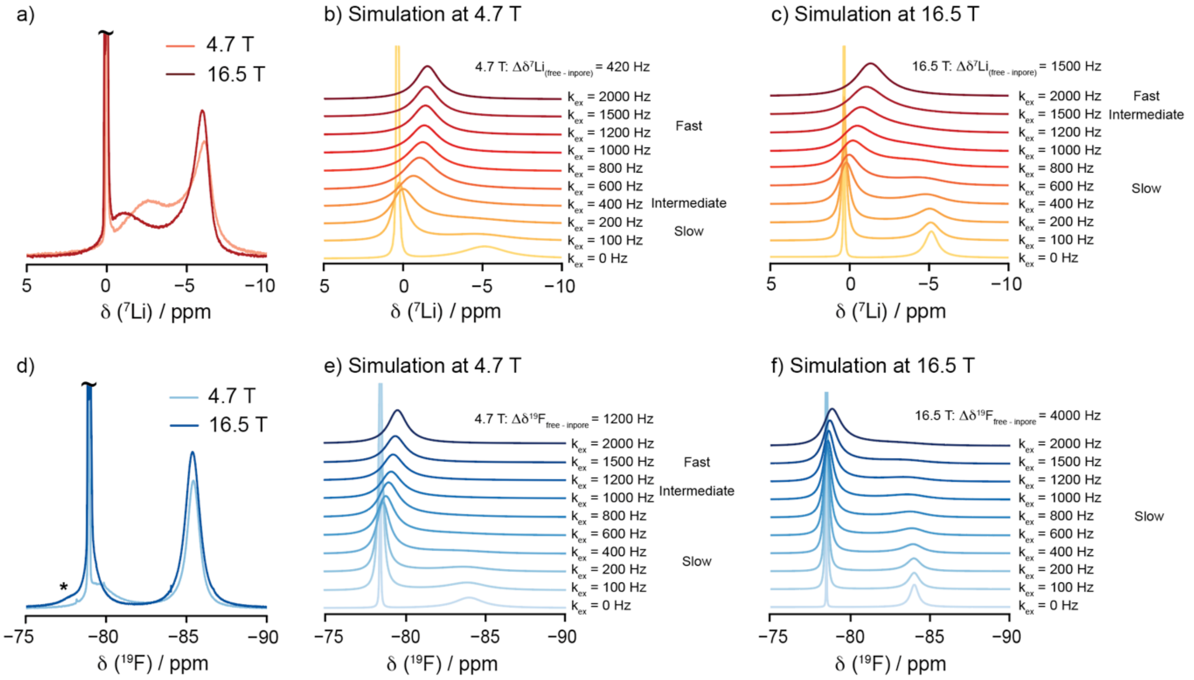

Figure S8. *a) ^{7}Li and d) ^{19}F NMR spectra of a YP-50F carbon sample saturated with 1 M LiTFSI electrolyte acquired at static magnetic field strengths of 4.7 T and 16.5 T at 295 K. Asterisks (*) denotes the spinning side band (MAS=5 kHz) at 16.5 T magnetic field strength. Simulated spectra of the exchange resonances at two magnetic field strengths: 4.7 and 16.5 Tesla, for ^{7}Li (b, c) and ^{19}F (e, f) at a range of exchange rates with $P_{ex-pore}$: $P_{in-pore}$ of 1.5:1 and 6:1 for ^{7}Li and ^{19}F, respectively.*

To further substantiate the assignment of the exchange peak, NMR spectra of a saturated carbon sample are compared at two different magnetic field strengths of 16.5 and 4.7 T (Figure S8). The exchange rate of Li^+ and $TFSI^-$ ions inside the porous carbon is independent of the magnetic field strength, whereas the frequency separation (Δδ) between the free and in-pore environments scales linearly (in Hz) with the magnetic field strength, meaning that at a higher magnetic field strength more rapid exchange is required for coalescence.

At 16.5 T, the ^{7}Li in-pore resonance displays greater intensity compared to that at 4.7 T. Moreover, at 16.5 T the chemical shift of the exchange peak closely aligns with the chemical shift of the free electrolyte peak, suggesting that exchange rate does not reach the coalescence point yet. These observations are consistent with fewer ions being in the fast-intermediate exchange regime at the higher field strength. Similar trends are observed in the ^{19}F spectra. The advantage of the magnetic field-dependent experiment is that at the two magnetic fields the only variable is the frequency separation, whereas in the variable temperature experiment the population of in-pore ions might fluctuate as a function of temperature which is not taken into account in the exchange rate simulation. To aid the interpretation of the magnetic field-dependent experiment, the spectra evolutions of the ^{7}Li and ^{19}F exchange resonances are simulated at two magnetic field strengths as a function of exchange rates, shown in Figure S8. The same range of the exchange rates were simulated, and the exchange peak at the higher magnetic field is now consistent with exchange in a slower exchange rate regime in comparison to that seen at the lower magnetic field due to the greater frequency separation between free and in-pore resonances.

8) Determination of in-pore electrolyte concentration

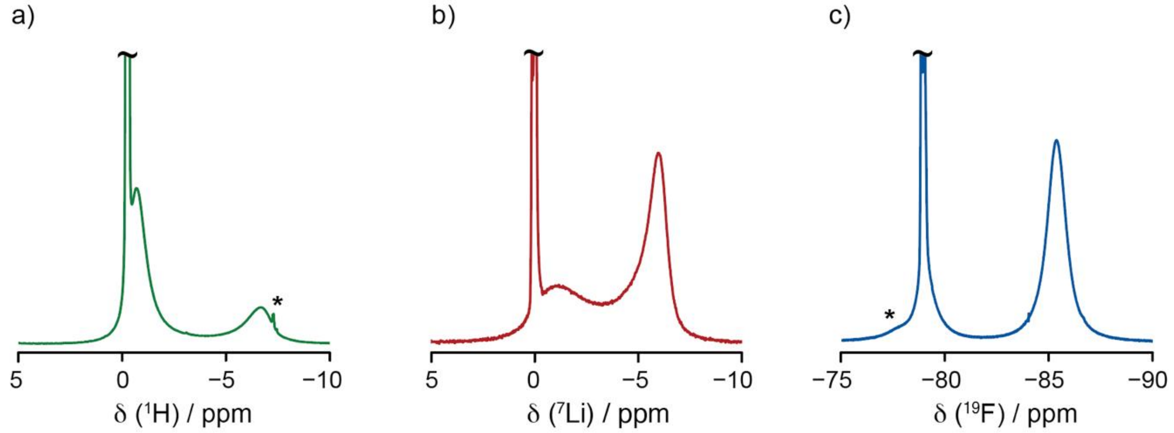

***Figure S9**. a)^{1}H, b) ^{7}Li, and c) ^{19}F MAS NMR spectra of a YP-50F carbon sample saturated with 1 M LiTFSI electrolyte acquired at a static magnetic field strength of 16.5 T at 295 K. Asterisks (*) in a) and c) denotes the spinning side band (MAS=5 kHz).*

The ^{1}H, ^{7}Li and ^{19}F NMR spectra of the carbon sample are acquired to determine the electrolyte ion concentration inside carbon pores (Figure S9). The ^{1}H NMR spectra show, similar to the ion exchange process seen in the ^{7}Li and ^{19}F NMR spectra, that there is a subset of water molecules undergoing rapid exchange in and out of the pores. The volume of in-pore water can then be determined by deconvolution of the ^{1}H NMR spectra to determine the fraction of water in the pores as outlined below.

The total volume and concentration of electrolyte inside the NMR rotor is known since 6 μL of 1 M LiTFSI aqueous electrolyte was added to 2 g of carbon for the sample analysed in Figure S9. Assuming that the density of water is the same inside and outside the pores, (i.e. the intensity of the water signal is directly proportional to the volume, independent of whether this signal comes from in- or ex-pore environments) the fraction of in-pore water (^{1}H) signal relative to the overall ^{1}H NMR signal intensities gives the volume of in-pore water. Since 22% of ^{1}H NMR signal in Figure S9a) comes from in-pore water, then the volume of in-pore water is 22% × 5 μL = 1.1 μL. Similarly, the moles of in-pore ions (^{7}Li, ^{19}F) are determined from the deconvolution of the in-pore NMR signals. For example, since 57% of the ^{7}Li NMR signal in Figure S9b) comes from in-pore Li^{+} and hence the number of moles of in-pore Li^{+} is 57% × (6 μL × 1 μmole/μL) = 3.4 μmol. The in-pore Li^{+} ion concentration ($[Li^{+}]_{in\text{-}pore}$) can then be calculated from the moles of in-pore ions ($n_{in\text{-}pore\ Li+}$) and the volume of in-pore water ($V_{in\text{-}pore\ water}$) by:

$$[Li^{+}]_{in\text{-}pore} = n_{in\text{-}pore\ Li+} / V_{in\text{-}pore\ water} \qquad \textit{Equation S3}$$

where both $n_{in\text{-}pore\ Li+}$ and $V_{in\text{-}pore\ water}$ are determined from the NMR quantification. Thus, the in-pore Li^{+} ion concentration is 3.4 μmol / 1.1 μL = 3.1 M. A similar calculation yields 3.3 ± 0.1 M for in-pore $TFSI^{-}$ ions. The numbers are noticeably higher than the ex-pore ion concentrations of 0.7 ± 0.2 M and 0.6 ± 0.1 M for Li^{+} and $TFSI^{-}$, respectively.

9) NMR concentration experiments

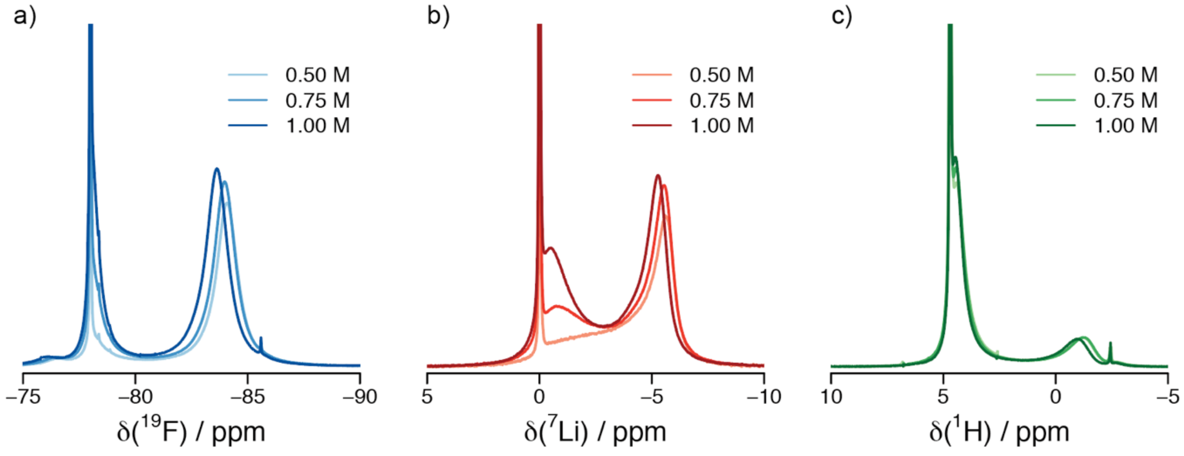

Figure S10. *The ^{19}F, ^{7}Li and ^{1}H spectra of YP-50F carbon saturated with 0.5 M, 0.75 M and 1 M of LiTFSI solution (loading volume of 1:3 w/v). The spectra were measured under a MAS rate of 5 kHz at 16.5 T.*

10) NICS calculations of phenol- and pyrone- functionalised carbon coronene rings

DFT geometry optimisations and NMR shift calculations were performed using the Gaussian 03 software package. YP-50F exhibits curved graphene-like sheets. As such, small graphene-like coronene rings are used as the model system to interpret the resonance frequencies of electrolyte ions adsorbed in the micropores of YP-50F carbon powder. The validity of using finite hydrocarbon units to model structural and magnetic properties of activated carbon has been demonstrated before.[4,5] Positions less than 3 Å away from the carbon planes were not considered as it is unlikely that molecules will be able to occupy these distances due to the van der Waals radii of the carbon and the probe ion. Nucleus-independent chemical shift (NICS) calculations predict the change in chemical shift of a hypothetical 'ghost atom' at a given position, based on the electron density near that position as obtained from a DFT calculation.[5] We calculated NICS for ghost atoms at a range of heights above the centre of a coronene ring, and above the edge of the ring, as demonstrated in Figure S11a-b.

NICS values were compared between pristine coronene and phenol-functionalised coronene (including neutral, protonated and deprotonated states) at the centre of the coronene plane, as shown in Figure S11c. The coronenes with neutral and protonated phenol functional groups exhibit a similar NICS to the pristine coronene of about -4.5 ppm, suggesting that the degree of aromaticity of the carbon domain is maintained on addition of phenol groups, i.e. little donation of the OH e^-, as expected. In addition, this calculated NICS of coronene (-4.5 ppm) is comparable in magnitude to the experimental measured $\Delta\delta$ value of about -5.5 ppm of YP-50F carbon in neutral 1M LiTFSI electrolyte. However, a small reduction of NICS is observed in the deprotonated form of phenol, indicating a reduced aromatic character in the carbon plane since filled O^- 2p atomic orbital easily interacts with empty π^* molecular orbital of coronene. The magnetic field at the edge of the ring, as shown in Figure S11d, resembles that at the centre of the ring but slightly smaller in magnitude, suggesting the electron density/aromaticity is slightly greater at the centre than at the edge of the carbon ring.

Furthermore, NICS calculations were performed on pyrone-functionalised coronene (including neutral and protonated forms) at the centre of the coronene plane, as shown in Figure S11e. In contrast to phenol, introducing pyrone at the edge of the coronene ring reduced the NICS from -4.4 to -2.5 ppm at 3 Å above the ring. Further protonation of pyrone enhances the electron density/aromaticity as evidenced by the increase of NICS from -2.5 to -3.9 ppm. The NICS at the edge of the ring shows a similar trend to the NICS at the centre of the ring (Figure S11f). Note that two edge sites were probed for pyrone, one site close to the C=O of pyrone and the other close to the C-O-C of pyrone. The NICS values at the two edge sites are the same which suggests that the electrons are delocalised across the pyrone. The increase of calculated NICS values for protonated pyrone in coronene contradicts to the hypothesis we had for the smaller NICS observed in YP-50F under acidic conditions. This discrepancy might be attributed to the over-simplified carbon model we used in the calculation which may not capture the effects such as curvature of the carbon sheets, solvent (H_2O) interactions with the carbon and the size of the carbon domain.

These calculations show that it does not seem to matter where on the ring the ghost atom sits, suggesting the ring currents are fairly uniformly across the ring, so the primary determinant for the shift is how many electrons are present in the aromatic ring. Thus, we can take the NICS principally as a measure of domain size (i.e. the coherence length of the delocalisation of carbons) and distance of ion from ring. The more complex results (functionalisation) are less clear and harder to interpret.

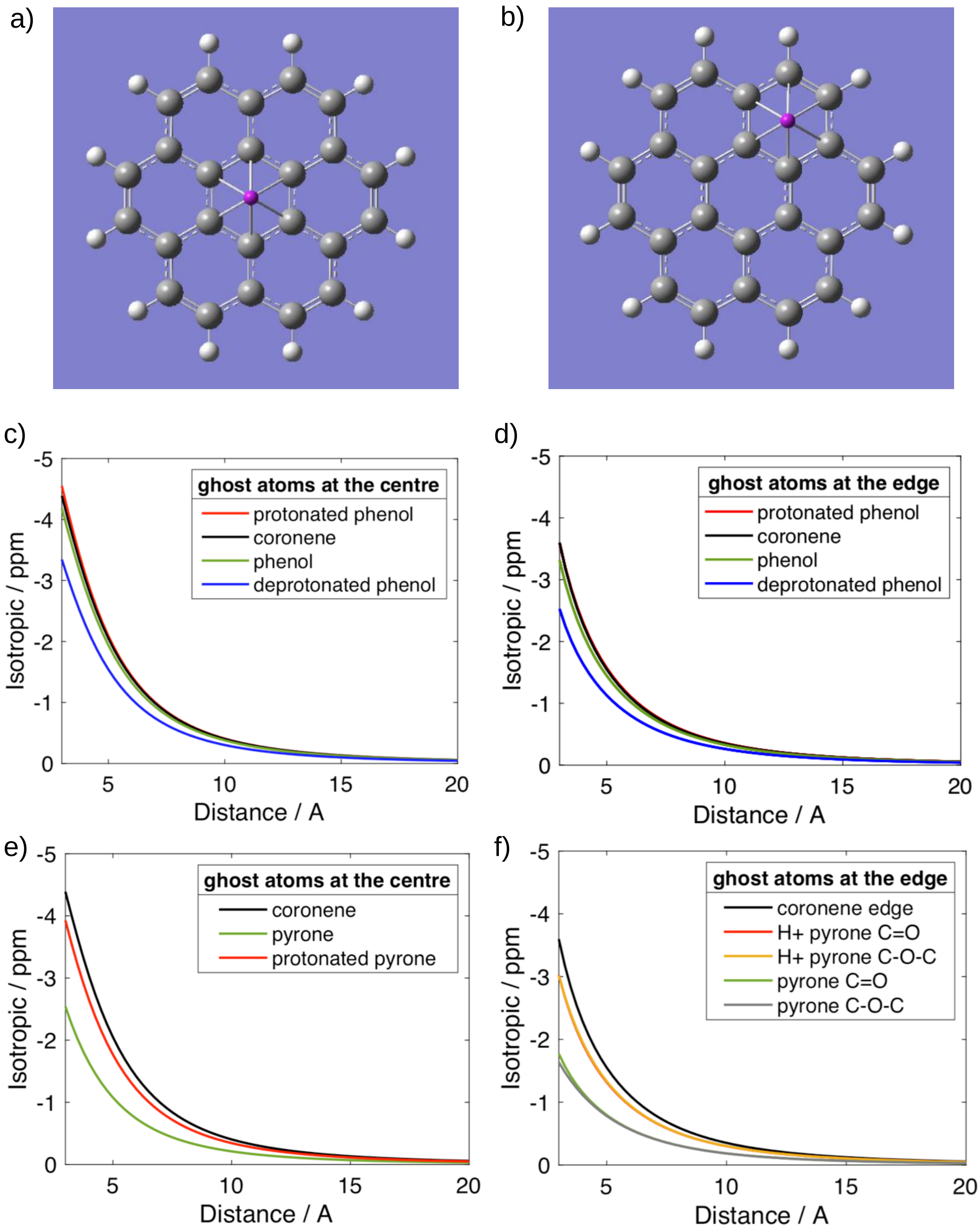

Figure S11. *The optimized structure of the coronene ring with ghost atoms sitting a) at the centre of the ring and b) at the edge of the ring. The calculated NICS of phenol-functionalised coronene with ghost atoms c) at the centre of the ring and d) at the edge of the ring. The calculated NICS of pyrone-functionalised coronene with ghost atoms e) at the centre of the ring and f) at the edge of the ring.*

References

1 A. V. Neimark, Y. Lin, P. I. Ravikovitch and M. Thommes, *Carbon N. Y.*, 2009, **47**, 1617–1628.

2 K. Märker, P. J. Reeves, C. Xu, K. J. Griffith and C. P. Grey, *Chem. Mater.*, 2019, **31**, 2545–2554.

3 J. R. Norris, *Chem. Phys. Lett.*, 1967, **1**, 333–334.

4 J. Vähäkangas, S. Ikäläinen, P. Lantto and J. Vaara, *Phys. Chem. Chem. Phys.*, 2013, **15**, 4634–4641.

5 A. C. Forse, J. M. Griffin, V. Presser, Y. Gogotsi and C. P. Grey, *J. Phys. Chem. C*, 2014, **118**, 7508–7514.
